# Melatonin Mitigates Doxorubicin‐Induced Cardiotoxicity by Targeting the USP18/PKM2 Axis Without Compromising Antitumor Efficacy

**DOI:** 10.1002/advs.78431

**Published:** 2026-10-26

**Authors:** Zhenyu Feng, Yijin Yang, Jie Bai, Pengqi Sun, Xuyang Sun, Jinshu Shang, Yang Liu, Zhen Ning, Ningning Zhang, Yun‐long Xia, Yunpeng Xie

**Affiliations:** ^1^ Institute of Cardiovascular Diseases The First Affiliated Hospital of Dalian Medical University Dalian P. R. China; ^2^ Department of Occupational and Environmental Health School of Public Health Dalian Medical University Dalian P. R. China; ^3^ Department of Hematology The First Affiliated Hospital of Dalian Medical University Dalian P. R. China; ^4^ Department of General Surgery The First Affiliated Hospital of Dalian Medical University Dalian P. R. China

**Keywords:** cardiotoxicity, doxorubicin, melatonin, PKM2, USP18

## Abstract

Doxorubicin (DOX) remains a widely used chemotherapeutic drug, yet its clinical value is constrained by pronounced cardiotoxicity. Although melatonin is known to protect the heart, how it counters DOX‐induced cardiotoxicity (DIC), and whether it interferes with chemotherapy, remains unclear. Here we set out to define how melatonin protects the heart during DOX treatment and to test whether this protection holds in tumor‐bearing animals. We used acute and chronic DIC mouse models together with a syngeneic melanoma‐bearing model, and combined transcriptomic profiling, pharmacological intervention, cardiomyocyte‐specific USP18 overexpression and knockout, co‐immunoprecipitation, mass spectrometry, ubiquitination assays, and PKM2 inhibitor treatment. Melatonin markedly improved cardiac function, an effect exerted mainly via the MT1/2 receptors. Mechanistically, DOX activated STAT1, which in turn drove myocardial USP18 upregulation. USP18 bound PKM2 and stabilized it through removing K33‐linked ubiquitin chains while favoring K27‐linked ubiquitination—thereby aggravating cardiac injury. Overexpressing USP18 specifically in cardiomyocytes worsened DIC and abolished the protection conferred by melatonin, whereas deleting USP18 or inhibiting PKM2 reproduced melatonin's benefits. Notably, in bearing melanoma or lymphoma, melatonin relieved DIC without weakening the antitumor activity of DOX. Together, these findings define a previously unrecognized cardioprotective pathway and provide preclinical support for melatonin as a safe adjunct therapy in cardio‐oncology.

## Graphical abstract

Melatonin engages MT1/MT2 receptors to suppress STAT1‐driven USP18 upregulation, thereby redirecting PKM2 ubiquitination from K33‐linked autophagic degradation toward K27‐linked nuclear translocation. This prevents PKM2‐mediated proinflammatory gene expression in the heart, alleviating doxorubicin‐induced cardiotoxicity without compromising antitumor efficacy, and positioning this pathway as a promising cardio‐oncology target.

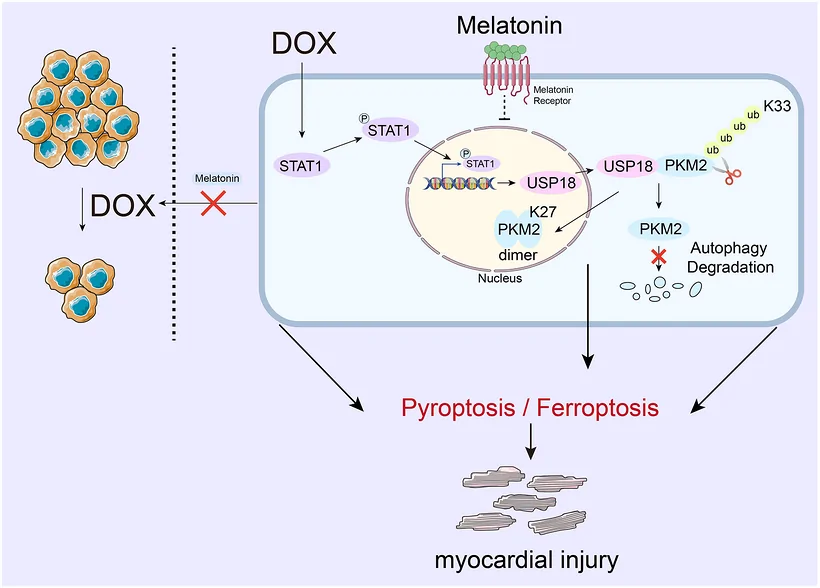

## 1 Introduction

Anthracyclines, particularly doxorubicin (DOX), constitute the cornerstone of modern cancer chemotherapy and are widely used for treating various malignancies, including breast cancer, lymphoma, sarcoma, and leukemia. However, this remarkable clinical efficacy is tempered by severe dose‐dependent cardiotoxicity [1]. In affected patients, this injury can appear as acute myocardial damage, evolve into chronic cardiac dysfunction, and in severe cases advance to irreversible dilated cardiomyopathy and congestive heart failure, which restricts prolonged anthracycline use and worsens the quality of life of cancer survivors [2]. The pathophysiology of doxorubicin‐induced cardiotoxicity (DIC) has been studied for decades and involves topoisomerase IIβ inhibition, oxidative stress, mitochondrial dysfunction, and activation of programmed cell death pathways [3, 4], the underlying molecular regulatory network remains incompletely understood. Notably, clinically available cardioprotective strategies that effectively mitigate DIC without compromising the antitumor efficacy of chemotherapeutic agents remain limited [5].

Currently, dexrazoxane, an iron chelator, represents the only FDA‐approved agent for mitigating DIC in clinical practice [6, 7]. However, concerns regarding its potential to attenuate the antitumor efficacy of DOX have prompted ongoing efforts to identify alternative cardioprotective strategies [8]. An ideal cardioprotective agent should selectively target cardiotoxic signaling pathways in cardiomyocytes without compromising the antitumor efficacy of doxorubicin. Addressing this challenge represents a critical unmet need in cardio‐oncology.

Melatonin (Mel), best known as a regulator of circadian rhythm, exerts antioxidant, anti‐inflammatory, anti‐apoptotic, and mitochondria‐protective actions in the cardiovascular system [9, 10, 11]. Preclinical studies have shown that melatonin protects against ischemia/reperfusion injury, diabetic cardiomyopathy, and drug‐induced cardiac injury [12, 13]. Part of this protection comes from direct scavenging of free radicals, but signaling through its receptors is thought to matter more. Melatonin engages two high‐affinity G protein‐coupled receptors, MT1 (Mel1a), and MT2 (Mel1b), which couple to downstream cardioprotective pathways [14, 15]. Melatonin also has roles in tumor biology, including inhibition of tumor growth through cell cycle modulation, metabolic reprogramming, and tumor microenvironment regulation. Given its dual cardioprotective and tumor‐regulatory properties, melatonin represents a promising candidate for preventing and treating DIC.

Ubiquitin‐specific protease 18 (USP18) is a member of the deubiquitinating enzyme family and has so far been studied mainly as a negative feedback regulator of interferon signaling. Recent studies have revealed that USP18 regulates cell proliferation, apoptosis, and inflammatory responses by deubiquitinating and stabilizing specific substrate proteins [16, 17]. Whether USP18 participates in drug‐induced myocardial injury, however, has not been examined. Pyruvate kinase M2 (PKM2), a rate‐limiting glycolytic enzyme frequently overexpressed in tumor cells [18]. Beyond its canonical metabolic function, PKM2 can translocate to the nucleus under cellular stress conditions, where it functions as a protein kinase or transcriptional coactivator to regulate key signaling nodes, including HIF‐1α and STAT3, thereby influencing cell fate decisions [19]. In the cardiovascular field, the role of PKM2 is gradually being elucidated [20]. It is unknown whether PKM2 is dysregulated in DIC or what upstream mechanisms might be involved. We also do not know whether USP18 and PKM2 functionally interact or whether they form a signaling axis in response to chemotherapeutic stress.

Building upon these knowledge gaps, this study aimed to systematically elucidate the molecular mechanisms underlying melatonin‐mediated protection against DIC and to evaluate its translational potential in cancer therapy settings.

## 2 Materials and Methods

### 2.1 Experimental Animals

Male C57BL/6 mice (8 weeks, 20–25g) were obtained from Liaoning Changsheng Biotechnology Co., Ltd. and housed under SPF conditions (22°C ± 2°C, 50% ± 5% humidity, 12h light/dark cycle). MT1 and MT2 knockout mice, and myocardial‐specific Usp18 conditional knockout mice (Myh6‐Cre; *Usp18^Fl/Fl^*), were generated by Cyagen Biosciences. DOX (Selleck, S1208) was given intraperitoneally at 20mg/kg cumulative dose (5mg/kg/week, 4 weeks). Melatonin (MCE, HY‐B0075, 20mg/kg/day), TEPP‐46 (TargetMol, T7214, 25mg/kg/day), and Compound 3K (TargetMol, T4170, 5mg/kg every 3 days) were administered intraperitoneally. All animal protocols were approved by Dalian Medical University (AEE21079) and complied with NIH guidelines. Mice were euthanized under anesthesia by cervical dislocation.

### 2.2 Cell Culture and Treatment

The AC16 (CL‐0790) cardiomyocyte cell line, HEK293T (293T) (CL‐0005) human embryonic kidney cell line, Panc02 (CL‐0736) mouse pancreatic cancer cells, and B16‐F10 (CL‐0319) mouse melanoma cells, EL‐4 (CL‐0078) mouse lymphoma cells, and PANC‐1 (CL‐0184) human pancreatic cancer cells were all purchased from Wuhan Procell Life Science & Technology Co., Ltd. AC16, 293T, and EL‐4 were cultured in DMEM high‐glucose with 10% FBS and 1% P/S. Panc02 and B16‐F10 were cultured in RPMI‐1640 with 10% FBS and 1% P/S. PANC‐1 was in DMEM/F12 with 10% FBS and 1% P/S. All cells were cultured at 37°C, 5% CO_2_. AC16 cells were treated with 0.5 µm DOX for 24 h to induce injury, with or without melatonin (MCE, HY‐B0075, 50 µm), luzindole (Selleck, S3584, 10 µm), or AGO (TargetMol, T1445, 10 µm) for 48 h. Vehicle‐treated cells served as controls. After the treatment, the cells were collected for subsequent experimental detection.

### 2.3 Echocardiographic Measurement

Cardiac function was measured using a Vevo 1100 system. Mice were anesthetized with 1.5%–2.0% isoflurane, placed supine at 37°C, and M‐mode images were obtained at the papillary muscle level. LVEF and LVFS were calculated.

### 2.4 Histological Staining

Heart tissues were fixed in 4% paraformaldehyde for 48 h, embedded in paraffin, and sectioned at 4 µm. Sections were stained with H&E (Solarbio, G1120), Masson's trichrome (Solarbio, G1340), WGA (Vector, RL‐1002, 25 µg/mL, 60min, dark), or DHE (Sigma, D7008, 10 µm, 10min, dark). Staining was performed according to the manufacturers' instructions. Sections were then mounted and imaged under light or fluorescence microscopy.

### 2.5 Immunohistochemical Staining

After the sections were de‐waxed, hydrated, and subjected to antigen retrieval, they were incubated at room temperature for 10 min with 3% H_2_O_2_ to block endogenous peroxidase activity. Goat serum was used for blocking. The primary antibodies against PCNA (ABclonal, lot number A0264) and Bax (ABclonal, lot number A19684) were incubated overnight at 4°C. HRP‐conjugated secondary antibody and DAB were used for development. Finally, hematoxylin was used for counterstaining.

### 2.6 Immunofluorescence Staining

#### 2.6.1 Cell sample

For cells grown on coverslips were fixed (with 4% PFA for 20 min), permeabilized (with 0.1% Triton X‐100 for 10 min), and blocked with goat serum. They were then incubated with the primary antibody against PKM2 (ABclonal, A20991), PKM1 (Proteintech, 15821‐1‐AP), PKM2 (Proteintech, 60268‐1‐Ig), LAMP1 (Beyotime, AG2482), PCNA (ABclonal, A0264), 8‐OHDG (GeneTex, GTX41980) and USP18 (ABclonal, A16739) at 4°C overnight.

#### 2.6.2 Tissue Samples

After dewaxing, rehydration, and antigen retrieval, 0.1% Triton X‐100 was applied for 10 min to achieve penetration, followed by blocking with goat serum. They were then incubated with the primary antibody against PKM2 (ABclonal, A20991), alpha Actinin (Invitrogen, MA5‐36095), Collagen III (Proteintech, 22734‐1‐AP), USP18 (ABclonal, A16739), and γ‐H2AX (Abcam, ab81299) at 4°C overnight.

After washing with PBS, the fluorescently labeled secondary antibody (Invitrogen) was incubated at room temperature in the dark for 1 h. The nuclei were stained with DAPI, and then images were captured.

### 2.7 Western Blot

Total protein was extracted with RIPA buffer and quantified by BCA. Samples (20 µg) were separated by SDS‐PAGE, transferred to PVDF membranes, blocked with 5% skimmed milk, and incubated with the following primary antibodies overnight at 4°C: PKM2 (ABclonal, A20991), PKM2 (Proteintech, 60268‐1‐Ig), PKM2 (CST, 4053S), PKM1 (Proteintech, 15821‐1‐AP), USP18 (CST, 4813S), USP18 (ABclonal, A16739), FLAG (Beyotime, AB3055), MYC (Proteintech, 60003‐2‐Ig), UB (Proteintech, 10201‐2‐AP), HA (Proteintech, 51064‐2‐AP), STAT1 (ABclonal, A12075), p‐STAT1‐S727 (ABclonal, AP0453), GPX4 (Abclonal, A1933), FTH (Abclonal, A19544), NLRP3 (Wanleibio, WL02635), and β‐actin (Affinity, T0022). After TBST washing, membranes were incubated with IRDye‐conjugated secondary antibodies and scanned using the Odyssey system. Band intensities were quantified with Image J.

### 2.8 Real‐Time Fluorescent Quantitative PCR (qPCR)

Total RNA was extracted using TRIzol, and cDNA was synthesized from 1 µg RNA. qPCR was performed with SYBR Green, using β‐actin as an internal control. Relative expression was calculated by 2^−ΔΔCt^ method (Table S1).

### 2.9 Transcriptomic Analysis

Total RNA was extracted from 9 human cell samples (3 groups: A, B, and C; n = 3 per group). Data analysis was conducted at Origingene (Origingene, Shanghai). Differentiated expressed genes (DEGs) were identified based on having a fold change greater than 1.5 or less than 2/3, as well as a p value of less than 0.05. Heatmap and volcano plot were performed using Xiantao (www.xiantao.love/). Strand‐specific cDNA libraries were constructed after mRNA enrichment and sequenced on the Illumina NovaSeq 6000 platform using the paired‐end 150 bp (PE150) strategy. Raw reads were filtered and trimmed using fastp (v0.23.0). The clean reads were aligned to the human reference genome GRCh38 (Ensembl release‐107) using HISAT2 (v2.2.1). Gene expression levels were quantified as raw read counts using StringTie (v2.2.1). Differential expression analysis between groups was performed using DESeq2 (v1.36.0). The complete raw gene count matrix is provided as Table S2 to ensure reproducibility.

### 2.10 DNA Pull‐Down Assay

Biotinylated DNA probes were immobilized on streptavidin beads and incubated with nuclear extracts at 4°C for 2–4 h. After high‐salt washing, bound proteins were eluted, and analyzed by Western blot.

### 2.11 In Vivo Antitumor Efficacy

Panc02 or B16‐F10 cells (1 × 10^6^ in 200 µL, 1:1 with Matrigel) were injected into the right flank of each mouse. When tumors reached 50–100 mm^3^, mice were randomized to receive DOX, melatonin. EL‐4 cells (5 × 10^4^) were injected into the right shoulder of each animal. Mice were randomized into six groups: control, melatonin (20mg/kg/day), 3K(5mg/kg/day), DOX(2mg/kg/day), melatonin+DOX, and 3K+DOX. Tumor volumes and body weights were monitored. Tumor volumes (V = L × W^2^/2) and body weights were recorded.

### 2.12 Transfection

When the AC16 cells and 293T cells reached a fusion degree of 60%, they were transfected using complete medium. 1 µg of the plasmid was mixed with Lipofectamine 8000 transfection reagent (Beyotime, C0533) in the recommended ratio as specified in the manual. Medium was replaced after 6 h, and cells were harvested at 24–48 h.

### 2.13 Co‐Immunoprecipitation (Co‐IP)

Cells were lysed in IP lysis buffer with protease inhibitors. Anti‐USP18 antibody and control IgG were incubated with Protein A/G magnetic beads at 4°C for 1–2 h to couple the antibody to the magnetic beads. After washing to remove unbound antibodies, the coupled magnetic beads were rotated with the above cell lysate supernatant at 4°C overnight. Beads were washed, and bound proteins were eluted and analyzed by Western blot.

### 2.14 Mass Spectrometry

Following SDS‐PAGE separation of cell lysates, the target protein bands were excised and subjected to in‐gel tryptic digestion. The resulting peptides were analyzed by nano‐LC‐MS/MS using a Q‐Exactive mass spectrometer. This service was provided by Beijing Huada Protein Research and Development Co., Ltd. Raw MS data (RAW files) were converted to MGF format via Proteome Discoverer 1.4 (Thermo Fisher Scientific) and searched against the UniProt human protein database (172 097 sequences) using the Mascot search engine (v2.3.01). Identifications were filtered at a false discovery rate of <1%. For instance, peptide coverage for PKM was 45%.

### 2.15 Cycloheximide, MG132, and Baf‐A1 assays

AC16 cells transfected with flag‐USP18 for 24 h were treated with cycloheximide (MCE, HY12320, 1 mm) at 0, 12, and 24 h before harvest to block protein synthesis. To define the degradation pathway of PKM2, MG132 (Selleck, S2619, 10 µm) or Baf‐A1 (TargetMol, T6740, 50 nm) was added 6 h prior to collection. Cell lysates were prepared and subjected to Western blotting to assess PKM2 protein levels, thereby evaluating the impact of USP18 on PKM2 stability.

### 2.16 Primary Cultures of Neonatal Rat Cardiomyocytes

The neonatal rat cardiomyocytes were isolated by enzymatic digestion. Under aseptic conditions, the hearts of Sprague‐Dawley pups aged 1–3 days were removed from the body, washed with ice‐cold PBS, and then the blood vessels and connective tissues were removed before the tissue was minced into fragments approximately 1 mm^3^ in size. These tissue fragments were placed in a solution containing 0.25% trypsin and digested by repeated shaking at 37°C; subsequently, each supernatant component was collected and neutralized with serum‐containing medium until complete digestion was achieved. After centrifuging the mixed cell suspension, it was resuspended in DMEM/F12 medium supplemented with 10% fetal bovine serum (FBS) and 1% P/S, and pre‐adherent cultured at 37°C, 5% CO_2_ for 90 min to promote the preferential adhesion of fibroblasts. The supernatant without adherent cells and rich in cardiomyocytes was collected, adjusted to the target cell density, and inoculated onto gel‐coated culture dishes for subsequent cultivation.

### 2.17 Primary Cultures of Adult Mouse Cardiomyocytes

After the mice were anesthetized with 1.5%–2.0% isoflurane, they were euthanized by cervical dislocation. The heart was quickly removed and EDTA buffer was perfused through the right ventricle to remove the residual blood. Then, the heart was sequentially perfused with EDTA buffer, perfusion buffer, and a digestion mixture containing type IV collagenase and Protease XIV. After digestion, the ventricular tissue was minced, filtered through a 100 µm sieve, and allowed to settle by gravity for 20 min. The myocardial cells and non‐myocardial cells were separated through three gravity settling cycles, and the cells were gradually re‐introduced with a stepwise buffer. The obtained myocardial cell precipitate was resuspended and inoculated onto a collagen‐coated culture dish. After incubation for 1 h, the free cells were removed by changing the culture medium, and the adherent cells were retained for subsequent experiments.

### 2.18 Biochemical Testing

The activities of lactate dehydrogenase, superoxide dismutase, and the content of malondialdehyde in serum were determined using commercially available kits (LDH: Elibscience, E‐BC‐K046‐M; SOD: Solarbio, BC5160; MDA: Solarbio, BC0025). All operations were strictly carried out in accordance with the operating procedures provided by each manufacturer.

### 2.19 Enzyme‐Linked Immunosorbent Assay (ELISA) for PKM2

The ELISA kit was used to quantitatively detect the circulating PKM2 concentrations in the serum of mice (ELK Biotechnology, ELK1516) and humans (ELK Biotechnology, ELK1514). The human samples were collected from 14 breast cancer patients who received liposomal doxorubicin chemotherapy treatment; all the subjects signed written informed consent after fully understanding the purpose of this study and the procedures. The research protocol was reviewed and approved by the Ethics Committee of the First Affiliated Hospital of Dalian Medical University (PJ‐KS‐KY‐2025‐373).

### 2.20 ChIP‐qPCR Experiment

Approximately 1 × 10^7^ cells were collected, washed with cold PBS, and cross‐linked with 1% formaldehyde at room temperature for 10 min. Then, glycine was used to terminate the reaction. The cells were lysed at 4°C using a lysis buffer for 30 min. Chromatin was fragmented by ultrasonication to generate DNA fragments mainly ranging from 200 to 700 bp. The supernatant after centrifugation was retained as the input group, while the rest was diluted with ChIP buffer and incubated with anti‐PKM2 antibody (Proteintech, 15822‐1‐AP, 3–5 µg) or control IgG (1 µL) in rotation overnight at 4°C. Protein A/G magnetic beads were added for further incubation for 2 h, followed by washing with elution buffer 1–4. The immune complexes were eluted with elution buffer and reverse cross‐linked with RNase A and proteinase K at 65°C overnight. The purified DNA was detected by qPCR using SYBR Green, and enrichment calculation was performed using the 2^−ΔΔCt^ method with the input group as the reference (Table S3).

### 2.21 Nuclear‐Cytoplasmic Fractionation

Cytoplasmic and nuclear fractions were isolated using a nuclear‐cytoplasmic separation kit (Solarbio, EX1470) according to the manufacturer's instructions. Briefly, harvest 5 × 10^6^ cells, wash with PBS, and resuspend in 200–300 µL of pre‐chilled Buffer A containing protease and phosphatase inhibitors. Gently incubate at 4°C with gentle shaking for 10–30 min. Centrifuge the lysate at 4°C and 2000×g for 5 min; collect the supernatant as the cytoplasmic fraction. Wash the pellet with PBS, then centrifuge again at 5000×g for 5 min. Resuspend the resulting pellet in 80–200 µL of inhibitor‐containing Buffer B, vortex for 15 s, and incubate at 4°C with shaking for 30–40 min. Finally, centrifuge at 4°C and 12 000×g for 10 min; collect the supernatant as the nuclear fraction.

### 2.22 Statistical Analysis

Statistical analyses were performed using GraphPad Prism 10.0, with all data presented as mean ± SD. Normality and variance homogeneity were assessed by Shapiro‐Wilk and Brown‐Forsythe tests, respectively. Intergroup differences between two groups were evaluated using an unpaired two‐tailed Student's t‐test. For comparisons involving more than two groups, one‐way or two‐way ANOVA was employed, followed by Tukey's post hoc test. Repeated‐measures two‐way ANOVA coupled with Sidak's multiple‐comparison test was applied to analyze tumor growth curves. Survival data were compared using the log‐rank (Mantel‐Cox) test. A two‐sided P‐value < 0.05 was considered statistically significant.

## 3 Results

### 3.1 Melatonin Protects Against Both Acute and Chronic Doxorubicin‐Induced Cardiomyopathy in Mice

To evaluate the therapeutic potential of Mel (Figure 1A) against DOX‐induced cardiotoxicity (DIC), we first established a chronic mouse model. Mice received intraperitoneal injections of Mel for 1 week, followed by DOX treatment over 4 weeks (Figure 1B). The survival curve revealed approximately 50% mortality in the DOX group, which was significantly reduced by Mel co‐treatment (Figure 1C). Compared to the control group, DOX‐treated mice exhibited a significant decrease in the HW/TL ratio, which was notably ameliorated by Mel (Figure 1D). Echocardiography showed that DOX significantly reduced EF% and FS%, both of which were significantly improved by Mel (Figure 1E,F). WGA staining demonstrated marked cardiomyocyte atrophy in the DOX group compared to controls, while Mel treatment significantly increased cardiomyocyte cross‐sectional area (Figure 1G). Masson staining indicated a significant increase in cardiac fibrosis upon DOX exposure, which was attenuated by Mel (Figure 1I). DHE staining showed a pronounced increase in oxidative stress in DOX‐treated hearts, which was suppressed by Mel (Figure 1K). qPCR analysis revealed that DOX significantly upregulated mRNA expression of the heart failure marker Bnp, the oxidative stress marker Nox2, and the fibrosis marker Collagen III. Co‐treatment with Mel significantly downregulated these markers (Figure 1H,J,L). Serum analysis showed that DOX increased MDA levels and decreased SOD activity, effects that were reversed by Mel (Figure 1M). Finally, LDH levels, an indicator of cell damage, were significantly elevated by DOX and reduced by Mel (Figure 1N). We also investigated the effect of Mel on acute DIC induced by a single high‐dose DOX injection (20 mg/kg) (Figure S1A). Echocardiography (Figure S1B), WGA staining (Figure S1C), LDH assay (Figure S1D), DHE staining (Figure S1E), Masson staining (Figure S1F), qPCR for Bnp, Collagen III, and Nox2 (Figure S1G), and serum SOD/MDA assays (Figure S1H) collectively demonstrated that Mel protects against acute DOX‐induced cardiac dysfunction. Furthermore, to assess the therapeutic potential of Mel, we administered Mel after initiating DOX treatment (Figure S2A). Survival analysis (Figure S2B), HW/TL ratio (Figure S2C), WGA staining (Figure S2D), Masson staining (Figure S2F), DHE staining (Figure S2H), qPCR for Bnp, Collagen III, and Nox2 (Figure S2E,G,I), serum SOD/MDA (Figure S2J), and LDH assays (Figure S2K) all confirmed that Mel treatment significantly reduces DIC. At the same time, we demonstrated in cardiomyocytes in vitro that DOX can stimulate DNA damage in cardiomyocytes, while Mel can inhibit DOX‑induced DNA damage (Figure S3).

**FIGURE 1:**
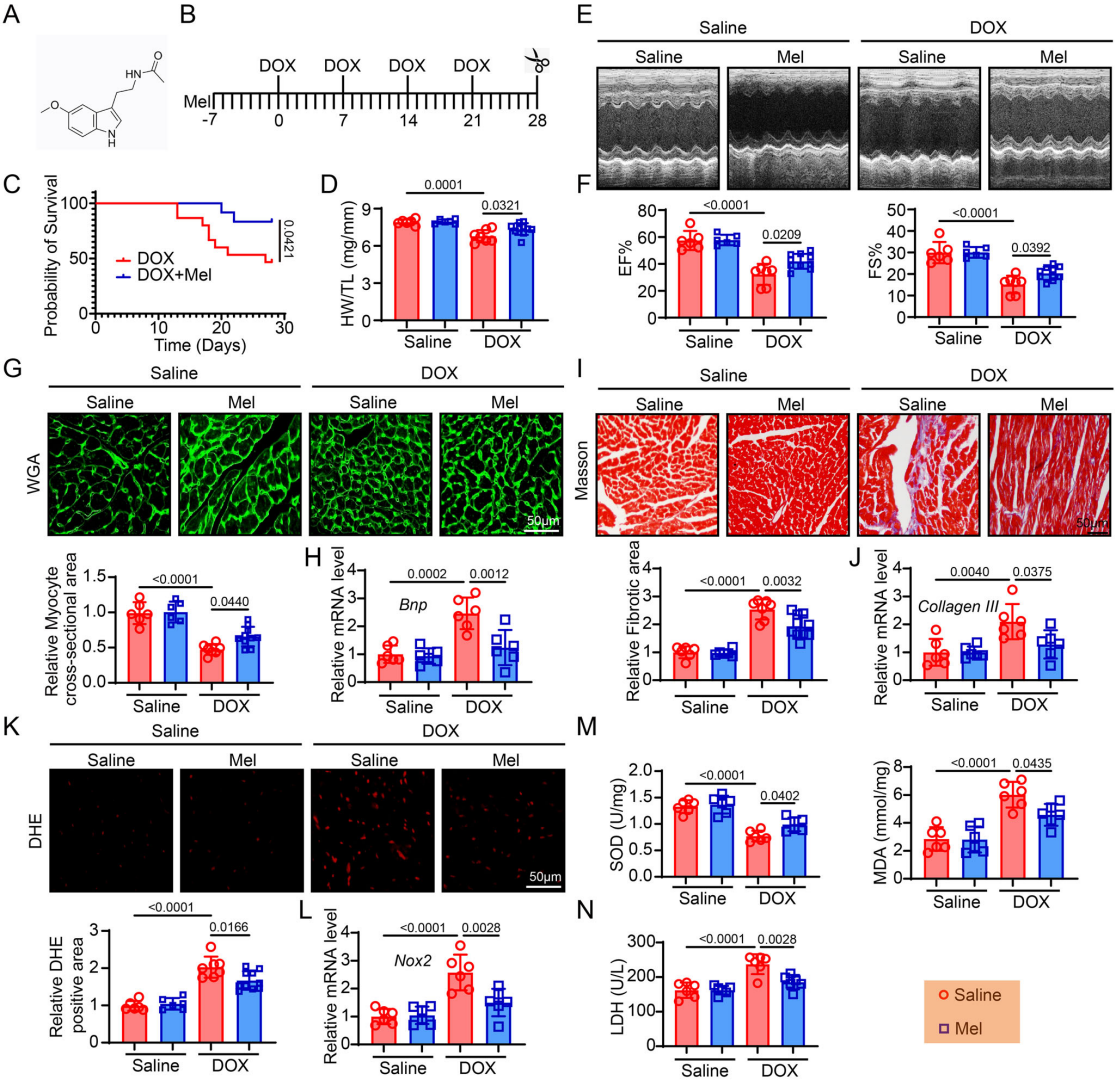
Melatonin protects against chronic doxorubicin‐induced cardiomyopathy. (A) Chemical structure of melatonin. (B) Schematic diagram of the experimental protocol for the chronic DIC model. (C) Kaplan–Meier survival curves of mice treated with DOX or DOX+Mel. (D) HW/TL ratio. (E) Representative echocardiographic images. (F) Quantification of EF% and FS%. (G) Representative WGA staining and quantification of cardiomyocyte cross‐sectional area. (H) qPCR analysis of *Bnp* mRNA expression. (I) Representative Masson and quantification of fibrotic area. (J) qPCR analysis of *Collagen III* mRNA expression. (K) Representative DHE staining and quantification of oxidative stress. (L) qPCR analysis of *Nox2* mRNA expression. (M) Serum levels of SOD and MDA. (N) Serum LDH levels.

### 3.2 Melatonin Inhibits DOX‐Induced Myocardial Injury via Its Membrane Receptors MT1 and MT2

Having shown that melatonin (Mel) limits DOX‐induced myocardial injury, we asked whether this protection depends on melatonin receptors, using MT1‐ or MT2‐deficient mice; all four groups received DOX. In DOX‐treated hearts, Mel enlarged cardiomyocyte cross‐sectional area on WGA staining, but this effect was lost in both the Mel+MT1 and Mel+MT2 groups, in which cardiomyocytes remained atrophic (Figure 2A). The same pattern held for fibrosis: Masson staining showed that Mel reduced collagen deposition relative to DOX alone, whereas loss of either receptor restored the fibrotic phenotype (Figure 2C). DHE staining gave a concordant result for oxidative stress—Mel lowered ROS accumulation, and deleting MT1 or MT2 abolished this reduction (Figure 2E). Myocardial mRNA levels of BNP, Collagen III and Nox2 followed suit: they fell with Mel treatment and rose again when either receptor was absent (Figure 2B,D,F). Serum biochemistry told the same story. Mel decreased MDA and LDH while restoring SOD activity, and in the Mel+MT1 and Mel+MT2 groups these indices reverted toward the DOX‐only pattern (Figure 2G,H).

**FIGURE 2:**
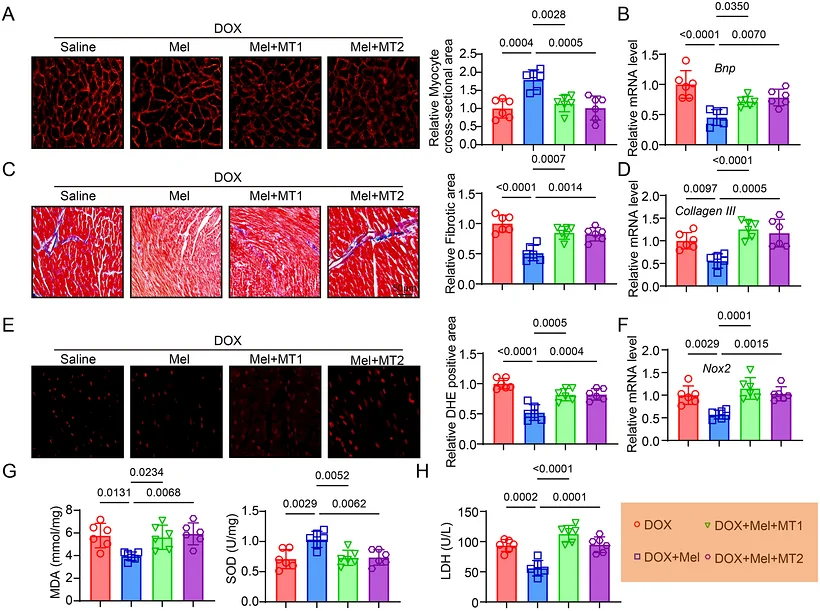
Melatonin inhibits DOX‐induced myocardial injury via MT1 and MT2 receptors. (A) Representative WGA staining and quantification of cardiomyocyte area. (B) qPCR analysis of *Bnp* mRNA. (C) Masson's trichrome staining and fibrosis quantification. (D) qPCR analysis of *Collagen III* mRNA. (E) DHE staining and quantification of oxidative stress. (F) qPCR analysis of *Nox2* mRNA. (G) Serum SOD and MDA levels. (H) Serum LDH levels.

### 3.3 USP18 is Identified as a Key Downstream Target Mediating Melatonin's Cardioprotection

In search of downstream effectors of Mel, we transcriptomically profiled AC16 cardiomyocytes exposed to DOX with or without Mel. Focusing on the ubiquitin‐specific protease family, heatmap and volcano plot analyses showed that DOX upregulated USP18 and USP11 while downregulating USP34, USP25, USP40, and USP13 relative to controls; among these genes, only USP18 returned toward baseline when Mel was added (Figure 3A–C). DOX stimulation likewise raised USP18 protein abundance in both AC16 cells and NRCMs (Figure 3D,E). Immunofluorescence in NRCMs confirmed this increase and showed that the induced USP18 was distributed across the cytoplasm and the nucleus (Figure 3F). At the mRNA level, DOX strongly induced USP18 transcription, and adding Mel largely abolished this induction (Figure 3G).

**FIGURE 3:**
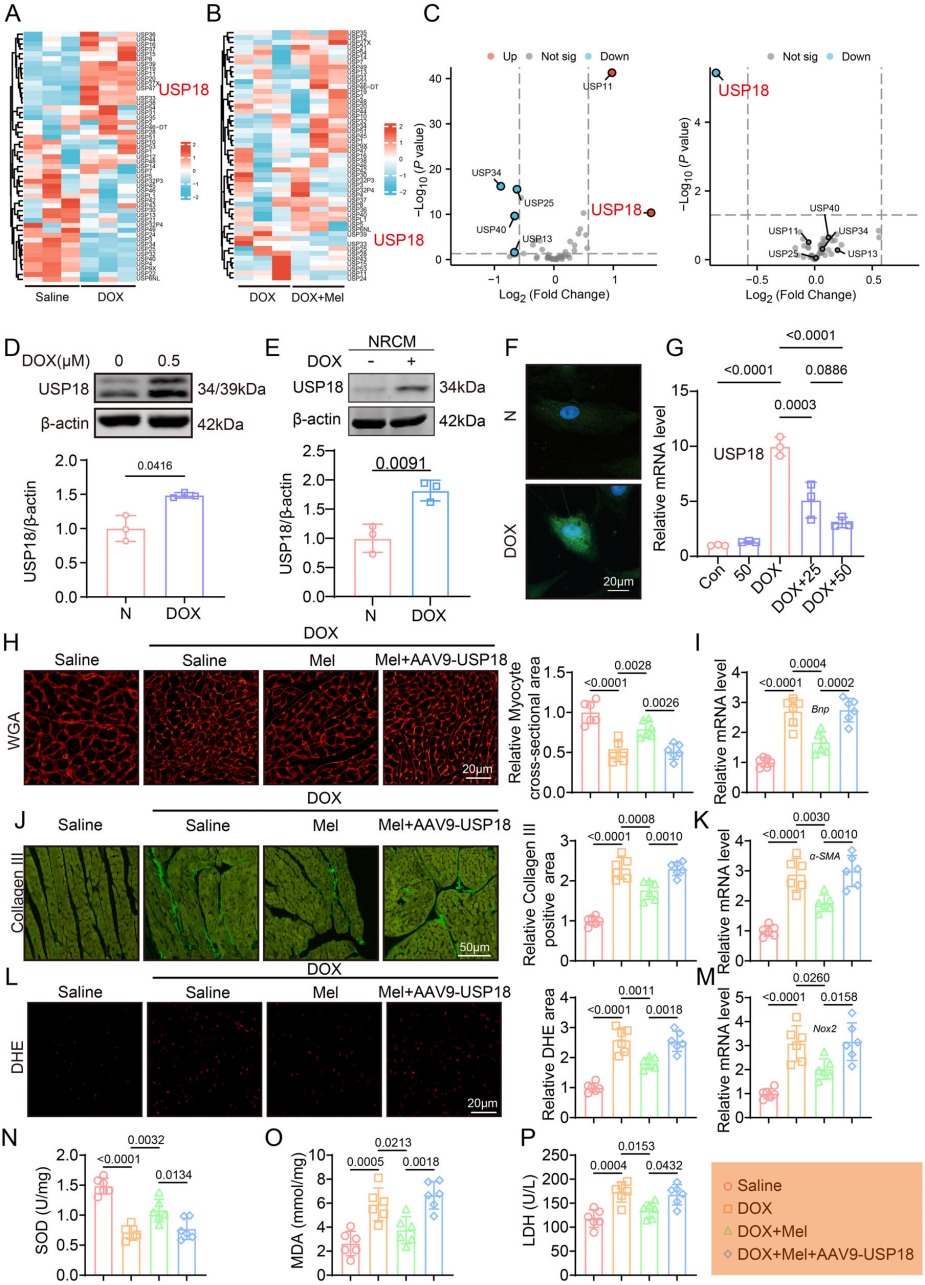
USP18 is identified as a key downstream target mediating melatonin's cardioprotection. (A) Heatmap showing differentially expressed USP family genes in AC16 cells. (B) Volcano plot of USP genes (DOX vs. Saline). (C) Volcano plot of USP genes (DOX+Mel vs. DOX). (D, E) Western blot analysis of USP18 protein in AC16 and NRCMs after DOX treatment. (F) Immunofluorescence staining of USP18 (green) in NRCMs; nuclei stained with DAPI (blue). (G) qPCR analysis of *USP18* mRNA in cardiomyocytes treated with DOX or Mel. (H) WGA staining and quantification of cardiomyocyte area in mice treated with DOX, DOX+Mel, or DOX+Mel+AAV9‐USP18. (I) qPCR of *Bnp* mRNA. (J) IF of Collagen III staining and quantification. (K) qPCR of *α‐SMA* mRNA. (L) DHE staining and quantification of oxidative stress. (M) qPCR of *Nox2* mRNA. (N, O) Serum SOD and MDA levels. (P) Serum LDH levels.

To test the functional role of USP18 in vivo, we generated a cardiomyocyte‐targeted USP18‐overexpressing adeno‐associated virus (AAV9‐cTNT‐USP18) and delivered it via the tail vein; DOX treatment began 1 week later and tissues were harvested after 4 weeks. qPCR, western blot and immunofluorescence verified robust USP18 overexpression at both mRNA and protein levels (Figure S4A–C). Forced USP18 expression worsened every aspect of the DIC phenotype—survival declined further (Figure S4D), injury‐marker transcripts rose (Figure S4E), EF% dropped (Figure S4F), and histology showed more vacuolization and inflammation (H&E, Figure S4G), more fibrosis (Masson, Figure S4H upper; Collagen III immunofluorescence, Figure S4H lower), smaller cardiomyocytes (WGA, Figure S4I), and stronger oxidative stress (DHE, Figure S4J).

We next combined DOX, Mel, and AAV‐USP18 to ask whether USP18 overexpression can override Mel's protection. It did. Mel preserved cardiomyocyte size in DOX‐treated mice, but forcing USP18 expression shrank cardiomyocytes again to near DOX‐only levels (WGA, Figure 3H). Collagen III immunostaining followed the same reversal: Mel reduced the DOX‐induced signal, and USP18 overexpression restored it (Figure 3J). DHE fluorescence told an identical story for oxidative stress, with Mel lowering the signal and USP18 overexpression bringing it back (Figure 3L). Transcripts of BNP, NOX2, and α‐SMA mirrored these histological changes—suppressed by Mel, re‐elevated when USP18 was overexpressed (Figure 3I,K,M). Serum indices behaved consistently: the fall in MDA and the recovery of SOD activity produced by Mel were both reversed by USP18 overexpression (Figure 3N,O), as was the reduction in circulating LDH (Figure 3P). In cultured cardiomyocytes, Mel attenuated DOX‐induced DNA damage, whereas USP18 knockdown provided additional attenuation of this damage (Figure S5).

### 3.4 Cardiomyocyte‐Specific Deletion of USP18 Directly Alleviates Doxorubicin‐Induced Cardiotoxicity

Having linked melatonin's protection to USP18 regulation, we asked whether USP18 by itself drives DIC pathogenesis. To this end we generated cardiomyocyte‐specific Usp18 knockout mice (Figure S6) and subjected them to the 4‐week DOX regimen before echocardiography and tissue collection. Deleting Usp18 markedly lowered DOX‐associated mortality (Figure 4A). The fall in EF% seen after DOX was partly rescued in Usp18 cKO hearts (Figure 4D). H&E staining showed that the vacuolization provoked by DOX was much less prominent in knockout hearts (Figure 4E), and both Masson staining and Collagen III immunofluorescence showed reduced collagen deposition in the DOX+Usp18 cKO group (Figure 4F). WGA staining confirmed that cardiomyocytes, which shrank after DOX in control mice, retained a larger cross‐sectional area in the DOX+Usp18 cKO group (Figure 4G). Oxidative stress, assessed by DHE staining and Nox2 qPCR, was strongly induced by DOX in controls but remained largely suppressed in knockout hearts (Figure 4H). Likewise, DOX‐induced upregulation of the inflammatory marker Il‐1β was blunted in Usp18 cKO mice (Figure 4B,C).

**FIGURE 4:**
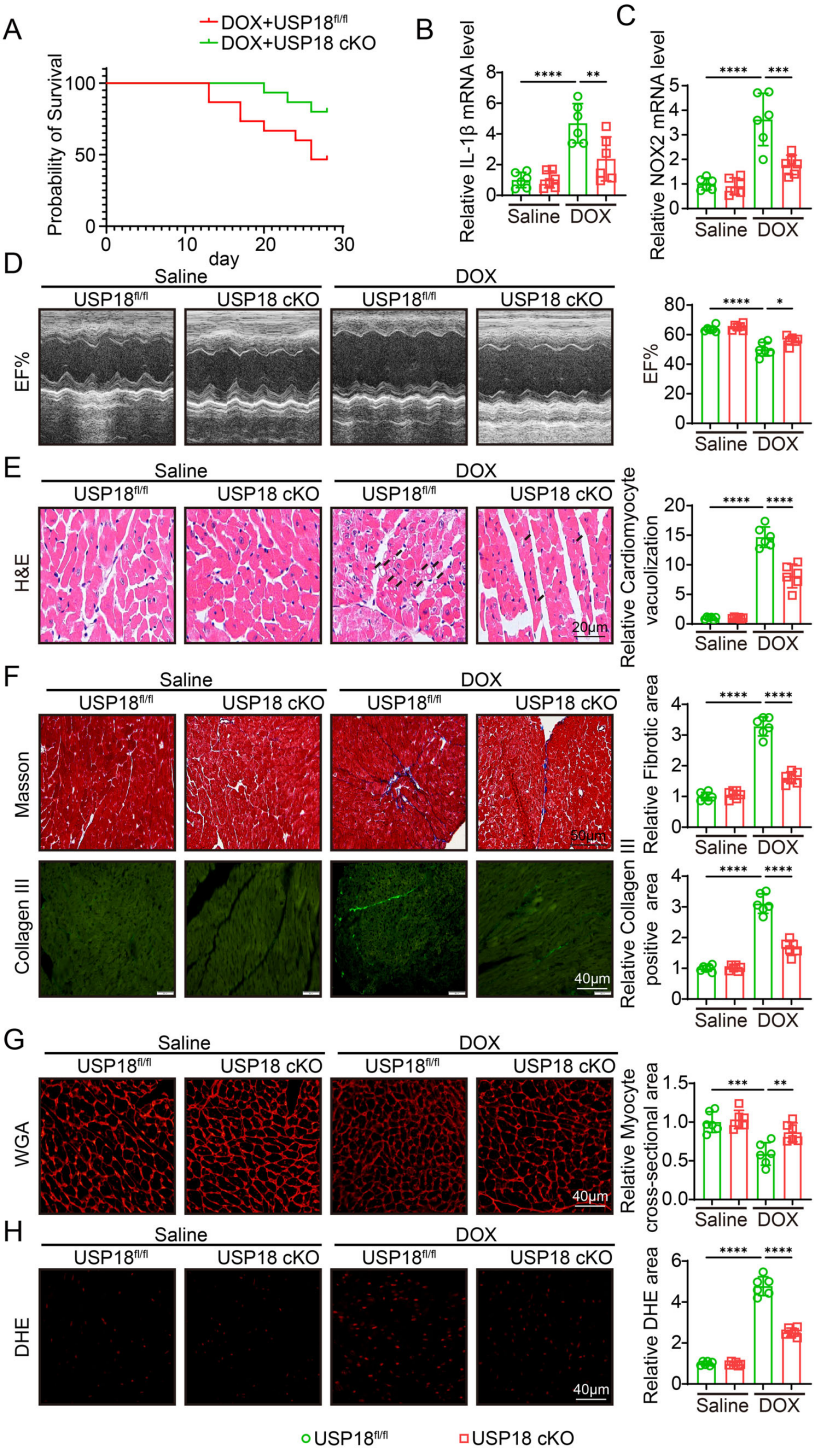
Cardiomyocyte‐specific deletion of USP18 directly alleviates doxorubicin‐induced cardiotoxicity. (A) Survival curves of WT and cardiomyocyte‐specific *Usp18* knockout mice treated with DOX. (B, C) qPCR analysis of *Il‐1β* and *Nox2* mRNA. (D) Echocardiographic EF%. (E) H&E staining and quantification of vacuolization. (F) Masson's trichrome staining (upper) and Collagen III immunofluorescence (lower) with quantification. (G) WGA staining and quantification of cardiomyocyte area. (H) DHE staining and quantification of oxidative stress.

### 3.5 Disruption of the Melatonin‐USP18 Axis Protects the Heart without Compromising the Antitumor Efficacy of Doxorubicin in Tumor‐Bearing Mice

To assess whether targeting the melatonin‐USP18 axis is translationally feasible, we turned to a syngeneic tumor‐bearing model, assigning melanoma‐bearing mice to four groups: Tumor alone, Tumor+DOX, Tumor+DOX+Mel, and Tumor+DOX+Usp18 cKO. Relative to Tumor+DOX, EF% was significantly better preserved in both the Mel‐treated and the Usp18 cKO groups (Figure 5A). These two interventions likewise lessened cardiac fibrosis on Masson's trichrome staining (Figure 5B), reduced myocardial vacuolization on H&E (Figure 5C), and preserved cardiomyocyte cross‐sectional area on WGA staining (Figure 5D). Crucially, serial tumor measurements over the tumor‐bearing period showed indistinguishable growth curves among the Tumor+DOX, Tumor+DOX+Mel, and Tumor+DOX+Usp18 cKO groups (Figure 5E), and endpoint tumor volume and weight did not differ either (Figure 5F–H). PCNA immunohistochemistry supported this conclusion: DOX lowered tumor PCNA staining relative to untreated tumors, and neither Mel nor Usp18 deletion altered this reduction (Figure 5I).

**FIGURE 5:**
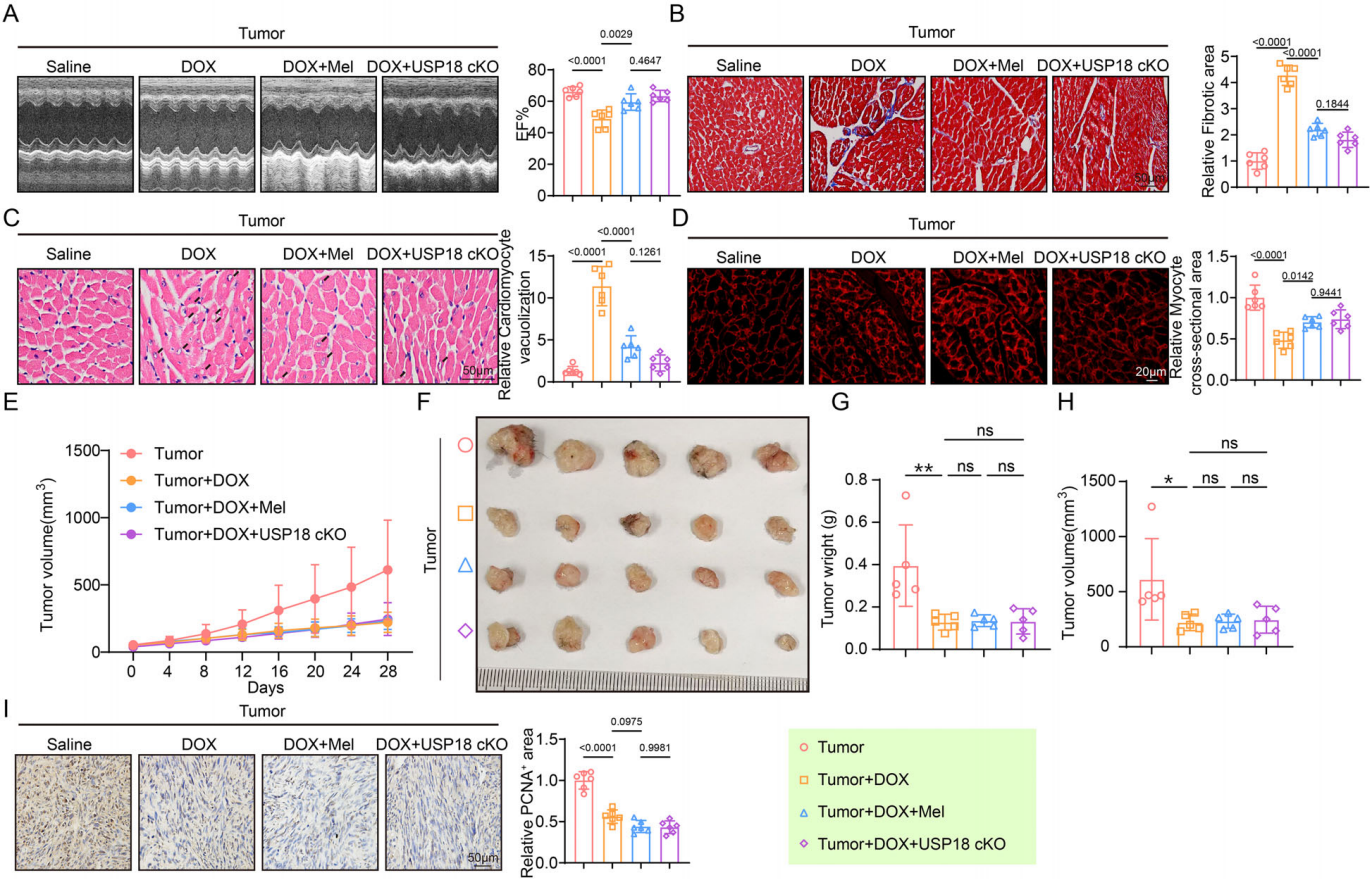
Disruption of the melatonin‐USP18 axis protects the heart without compromising antitumor efficacy in tumor‐bearing mice. Syngeneic melanoma‐bearing mice were assigned to four groups: Tumor only, Tumor+DOX, Tumor+DOX+Mel, and Tumor+DOX+*Usp18 cKO*. (A) Echocardiographic EF%. (B) Masson's trichrome staining and fibrosis quantification. (C) H&E staining for vacuolization score. (D) WGA staining and cardiomyocyte cross‐sectional area. (E) Tumor growth curves during the experimental period. (F–H) Tumor volume and weight at endpoint. (I) Immunohistochemical staining for PCNA and quantification.

Consistently, in a separate syngeneic melanoma‐bearing mouse model (Figure S7A), we confirmed that Mel treatment protected against DOX‐induced chronic myocardial injury, as evidenced by improved cardiac function (echocardiography, Figure S7B), reduced fibrosis (Masson staining, Figure S7C), and attenuated cardiomyocyte atrophy (WGA staining, Figure S7D). Concurrent measurement of tumor volume demonstrated that, while DOX treatment significantly reduced tumor volume compared to the control group, there was no significant difference in tumor volume between the DOX group and the DOX+Mel group (Figure S7E). In vitro, USP18 overexpression in tumor cells increased PCNA expression under DOX treatment, whereas USP18 knockdown further reduced PCNA expression (Figure S8A,B).

### 3.6 STAT1 Mediates DOX‐Induced USP18 Transcriptional Upregulation

To identify transcription factors binding to the USP18 promoter, we performed DNA pull‑down followed by mass spectrometry. As shown in Figure 6A, a specific protein band was detected only in the USP18 promoter probe group but not in the control probe group. This band was excised and subjected to MS analysis, which identified STAT1 as the top candidate (Figure 6A–C). DOX stimulation markedly increased STAT1 phosphorylation (p‐STAT1) in cardiomyocytes (Figure 6D,E), whereas the addition of mel inhibits the expression of p‐STAT1, but the expression of p‐STAT1 is significantly increased after further addition of the mel receptor inhibitor Luzindole (Figure 6F). Knockdown of STAT1 (945) significantly abrogated DOX‐induced USP18 mRNA expression (Figure 6G,H), whereas STAT1 overexpression further enhanced USP18 transcription (Figure 6I,J). Finally, we stimulated cardiomyocytes with DOX, the Mel receptor agonist agomelatine (AGO), and si‐STAT1, and found that Mel inhibited the expression of USP18, which was further reduced after the addition of AGO. After adding si‐STAT1, the expression of USP18 was further reduced (Figure 6K). These results demonstrate that DOX upregulates USP18 transcription via STAT1 activation, and that melatonin intervention suppresses STAT1 phosphorylation and subsequent USP18 expression.

**FIGURE 6:**
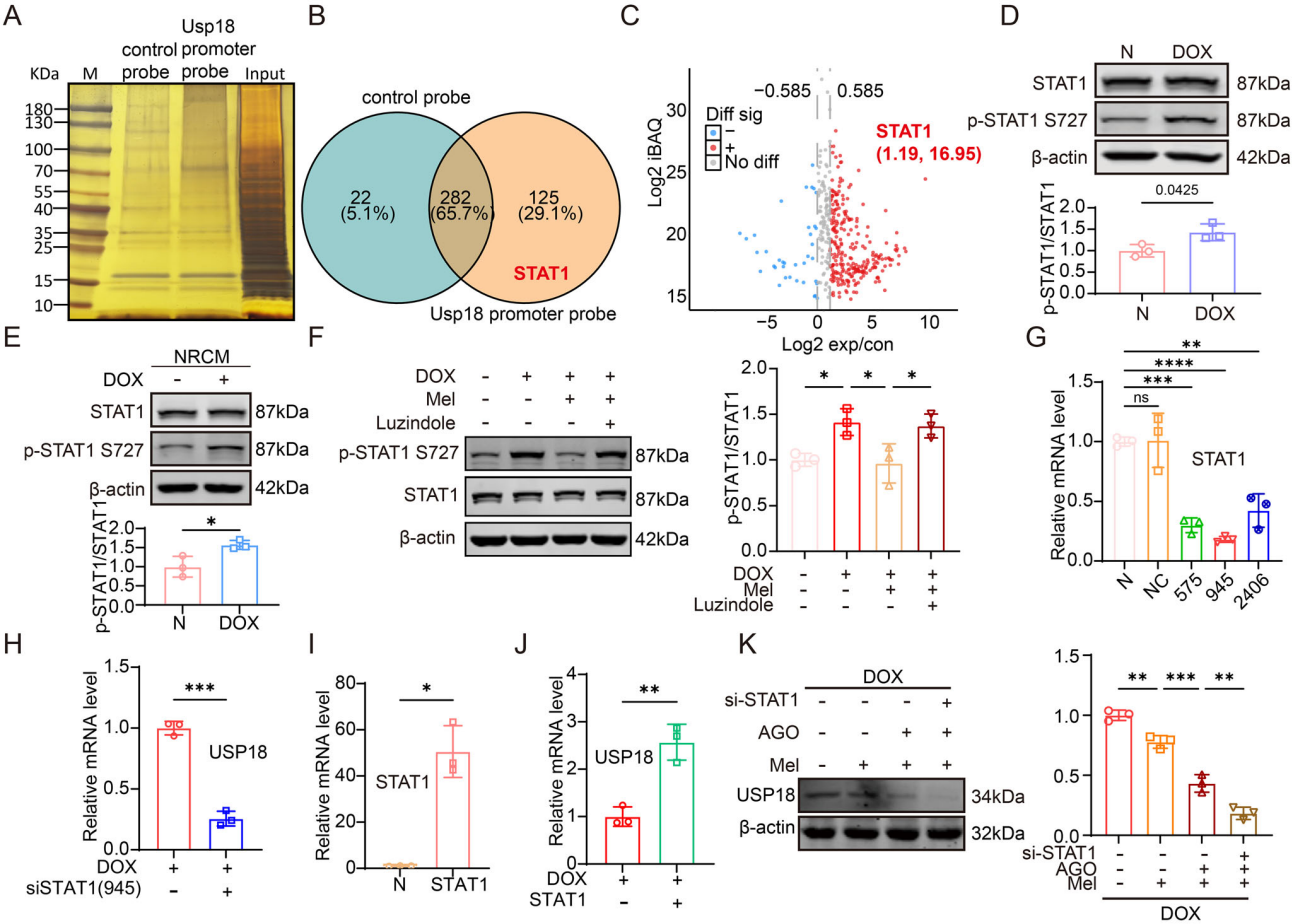
STAT1 mediates DOX‐induced USP18 transcriptional upregulation. (A–C) DNA pull‑down assay with the biotinylated Usp18 promoter probe. Nuclear extracts from cardiomyocytes were incubated with control probe (non‑biotinylated) or Usp18 promoter probe. Protein‑DNA complexes were pulled down with streptavidin beads, resolved by SDS‑PAGE, and visualized by silver staining. The specific band in the Usp18 promoter probe lane (arrow) was excised and subjected to mass spectrometry (MS). MS identified STAT1 as the top candidate transcription factor binding to the Usp18 promoter. (D, E) Western blot analysis of p‐STAT1 and total STAT1 in cardiomyocytes after DOX treatment. (F)  Western blot analysis of p‐STAT1 and total STAT1 in cardiomyocytes after DOX, Mel, and Luzindole treatment.  (G) qPCR of *STAT1* mRNA following STAT1 knockdown using three independent siRNAs (575, 945, and 2406). (H) qPCR of *USP18* mRNA after STAT1 Knockdown. (I) qPCR of *STAT1* mRNA following STAT1 overexpression. (J) qPCR of *USP18* mRNA after STAT1 overexpression. (K) Western blot analysis of usp18 in cardiomyocytes after DOX, AGO, and si‐STAT1 treatment.

### 3.7 USP18 Interacts with PKM2 and Inhibits its Ubiquitination

To find USP18‐interacting proteins, we immunoprecipitated USP18 from DOX‐stimulated cardiomyocytes and analyzed the precipitates by mass spectrometry; PKM emerged as a candidate substrate (Figure S9A). WB and IF showed that PKM1 barely changed after DOX in AC16 cells and NRCMs (Figure S9B–D), whereas PKM2 was strongly induced—in cardiomyocytes of DOX‐treated mice and in primary adult mouse cardiomyocytes alike (Figure S9E). In isolated adult mouse cardiomyocytes, immunofluorescence captured DOX‐driven nuclear translocation of PKM2 (Figure S9F). Two observations support the clinical relevance of this induction: serum PKM2 rose in patients after four cycles of liposomal doxorubicin relative to pre‐treatment values (Figure S9G), and the same increase occurred in mice after 4 weeks of DOX (Figure S9H). Co‐IP, molecular docking and immunofluorescence co‐localization each supported a direct interaction between USP18 and PKM2 (Figure 7A–C), and cycloheximide (CHX) chase assays showed that USP18 overexpression prolonged PKM2 protein half‐life (Figure 7D). In 293T cells expressing Flag‐USP18 together with Myc‐PKM2, the two proteins precipitated each other reciprocally with anti‐Flag and anti‐Myc antibodies (Figure 7E,F). When HA‐ubiquitin was co‐expressed with Myc‐PKM2 in 293T cells, USP18 overexpression lowered PKM2 ubiquitination, whereas USP18 knockdown intensified it (Figure 7G,H). In AC16 cells, Mel enhanced PKM2 ubiquitination, and this enhancement was reversed when USP18 was overexpressed (Figure 7I). Finally, truncation mapping in both 293T and AC16 cells localized the PKM2‐binding region of USP18 to amino acids 51–151 (Figure 7J–N).

**FIGURE 7:**
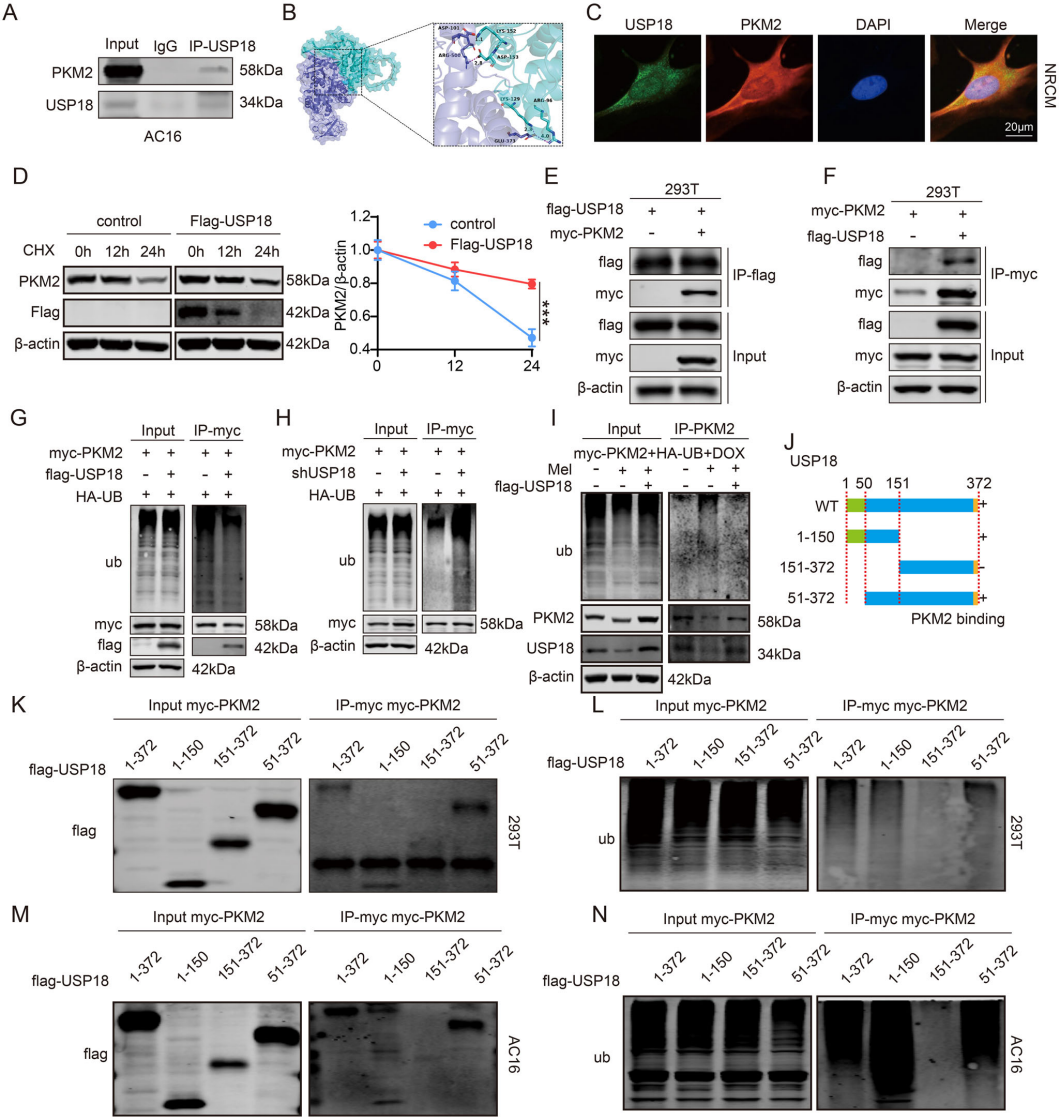
USP18 Interacts with PKM2 and Inhibits Its Ubiquitination. (A) Co‐IP was performed to detect the interaction between endogenous USP18 and PKM2 in AC16 cells. (B) Schematic diagram of molecular docking between USP18 and PKM2. (C) IF co‐localization assay was performed to detect the co‐localization of USP18 and PKM2 in NRCMs. (D) CHX chase assay was performed to detect the protein stability of PKM2 in cardiomyocytes with or without USP18 overexpression. (E, F) 293T cells were co‐transfected with Flag‐USP18 and Myc‐PKM2. Reciprocal co‐IP assays using anti‐Flag or anti‐Myc antibodies. (G, H) 293T cells were co‐transfected with Myc‐PKM2, HA‐Ubiquitin, and either Flag‐USP18 or shUSP18 to detect the effect of USP18 overexpression or knockdown on PKM2 ubiquitination. (I) AC16 cells were co‐transfected with Myc‐PKM2, HA‐Ubiquitin, and treated with Mel or Flag‐USP18 to detect the effect of USP18 overexpression on Mel‐induced PKM2 ubiquitination by WB. (J) Schematic diagram of USP18 truncation mutants. (K‐N) Co‐IP was performed in 293T and AC16 cells co‐transfected with Myc‐PKM2 and Flag‐tagged USP18 truncation mutants to map the PKM2‐binding domain on USP18.

### 3.8 USP18 Alters PKM2 Ubiquitination, Facilitating Nuclear Translocation and Inhibiting Lysosomal Degradation

To determine which ubiquitination linkage site of PKM2 is targeted by USP18 deubiquitination, we transfected constructs for K6, K11, K27, K29, K33, K48, and K63‐linked ubiquitin. The results showed that overexpression of USP18 significantly reduced K33‐linked ubiquitination (associated with autophagy) while significantly increasing K27‐linked ubiquitination (associated with nuclear translocation) (Figure 8A). Subsequent transfection with K27R and K33R mutants revealed that after transfection with these two mutants, the differences in ubiquitination levels were abolished (Figure 8B). Next, we stimulated cardiomyocytes with DOX, Mel, and USP18 plasmids, and extracted cytoplasmic and nuclear proteins. The results showed that there was no significant difference in cytosolic PKM2 protein among all groups. However, in the nucleus, compared with the control group, PKM2 expression was significantly increased in the DOX group; compared with the DOX group, PKM2 protein levels were markedly decreased after Mel treatment, while overexpression of USP18 significantly increased PKM2 expression (Figure 8C). Subsequently, we transfected K27 and K27R plasmids. Western blot and immunofluorescence results revealed that cytosolic PKM2 protein showed no significant differences among groups, whereas in the nucleus, compared with the K27 group, USP18 overexpression significantly increased PKM2 expression, while K27R transfection significantly decreased PKM2 protein levels, and USP18 overexpression failed to enhance PKM2 expression (Figure 8D,E). We then examined LAMP1 and PKM2 expression by immunofluorescence. The results demonstrated that USP18 overexpression suppressed LAMP1 expression and promoted PKM2 nuclear entry (Figure 8F), whereas USP18 knockdown enhanced LAMP1 expression and inhibited PKM2 nuclear translocation (Figure 8G). We treated cells with a proteasome inhibitor (MG132) and an autophagy inhibitor (Baf‐A1). The results showed that MG132 did not inhibit PKM2 degradation, whereas Baf‐A1 significantly inhibited PKM2 degradation, indicating that PKM2 degradation occurs primarily through the autophagy‐lysosome pathway (Figure 8H). To test whether nuclear PKM2 regulates inflammatory gene transcription, we performed ChIP‑qPCR in AC16 cells using anti‑PKM2 or IgG. In control cells, no PKM2 enrichment was detected at the IL1B, IL6, or IL18 promoters. DOX induced modest enrichment at IL1B and IL6, which was significantly enhanced by PKM2 overexpression, whereas IL18 showed no consistent enrichment, suggesting it is not a direct target. Thus, PKM2 directly occupies IL1B and IL6 promoters in DOX‑injured cardiomyocytes, and increased PKM2 boosts this regulatory function. (Figure 8I–K).

**FIGURE 8:**
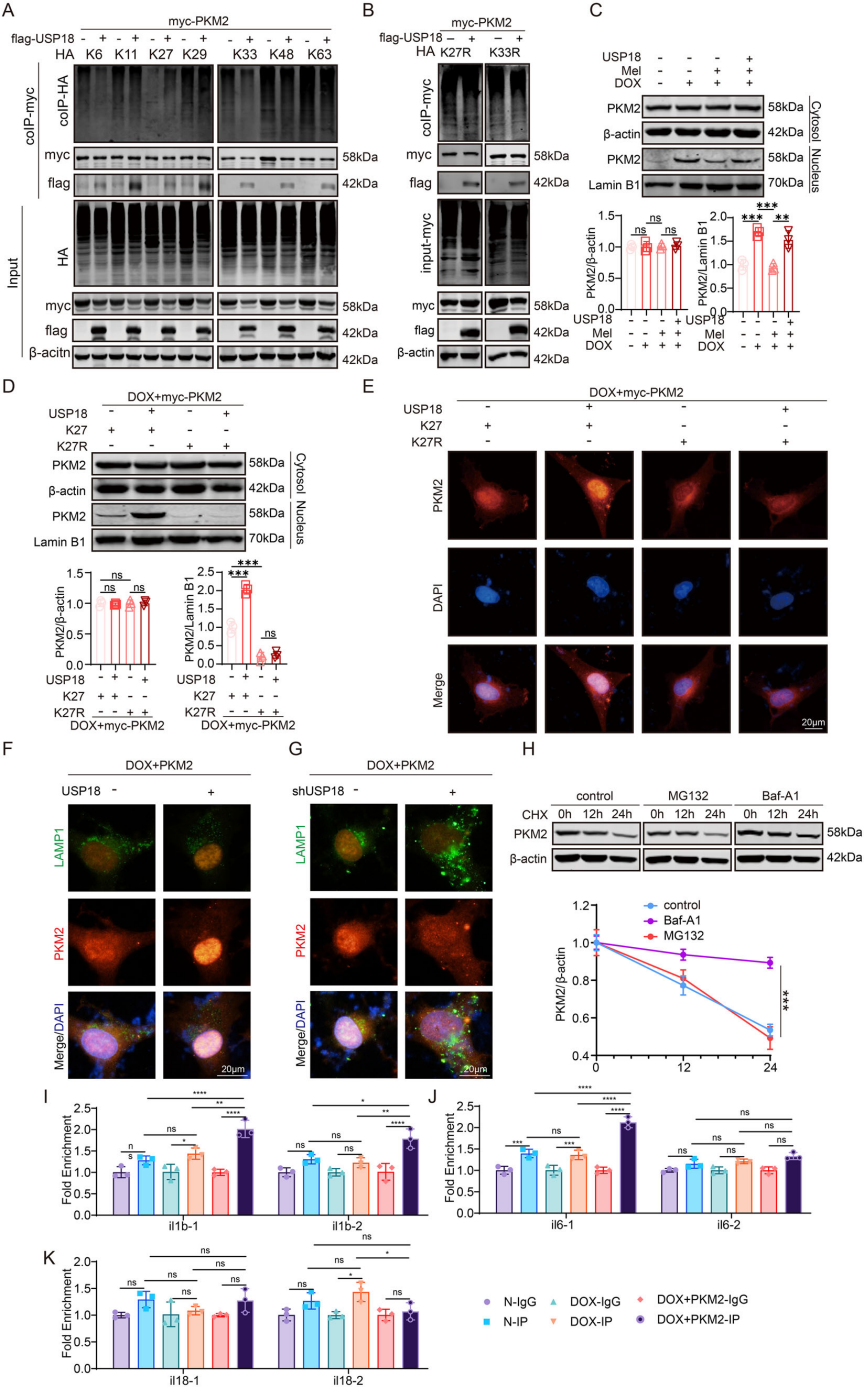
USP18 Alters PKM2 Ubiquitination, Facilitating Nuclear Translocation, and Inhibiting Lysosomal Degradation. (A) Ubiquitination assay was performed in 293T cells co‐transfected with Myc‐PKM2, Flag‐USP18, and HA‐tagged ubiquitin mutants to detect the ubiquitin linkage types regulated by USP18. (B) Ubiquitination assay was performed in 293T cells transfected with K27R and K33R ubiquitin mutants to confirm the specific ubiquitin linkage sites targeted by USP18. (C) Cardiomyocytes were stimulated with DOX, Mel, and USP18‐overexpressing plasmids; cytoplasmic and nuclear fractions were isolated to examine PKM2 subcellular distribution. (D, E) Cardiomyocytes were transfected with K27 or K27R plasmids together with USP18 overexpression, and PKM2 levels in cytoplasmic and nuclear fractions were detected by Western blot (D) and immunofluorescence (E). (F, G) Immunofluorescence staining of LAMP1 and PKM2 was performed in cardiomyocytes with USP18 overexpression (F) or knockdown (G) to evaluate lysosomal marker expression and PKM2 nuclear translocation. (H) Cardiomyocytes were treated with MG132 or Baf‐A1, and PKM2 protein levels were detected by Western blot to determine the degradation pathway of PKM2. (I–K) ChIP‐qPCR analysis of PKM2 occupancy at the *IL1B, IL6, and IL‐18* promoters using two independent primer pairs.

### 3.9 Pharmacological Inhibition of PKM2 Mimics Melatonin's Cardioprotection and Rescues Injury Induced by USP18 Overexpression

To test the therapeutic value of PKM2 blockade, mice received the PKM2 inhibitor 3K intraperitoneally for 1 week before the 4‐week DOX course. Relative to DOX alone, 3K treatment lessened cardiomyocyte atrophy (WGA, Figure S10A), fibrosis (Masson, Figure S10C), oxidative stress (DHE, Figure S10E), and γH2AX accumulation (immunofluorescence, Figure S10G), lowered myocardial Bnp, Nox2, and Collagen III transcripts (qPCR, Figure S10B,D,F), reduced serum LDH and MDA, and restored SOD activity (Figure S10H–J). PKM2 inhibition thus recapitulated Mel's protection against DOX‐induced myocardial injury. In an EL‐4 lymphoma‐bearing cohort treated with DOX, adding Mel or 3K did not weaken the antitumor activity of DOX—tumor volumes, tumor weights, and PCNA staining were comparable between the DOX and DOX+3K groups (Figure S11A–I)—indicating that PKM2 inhibition on its own also leaves antitumor efficacy intact. Conversely, the PKM2 agonist TEPP‐46, given intraperitoneally for 1 week before DOX, aggravated DOX‐induced myocardial injury (Figure S12A–F). To place PKM2 definitively downstream of USP18 within the melatonin signaling axis, we performed a rescue experiment with four groups: DOX, DOX+Mel, DOX+Mel+AAV9‐USP18, and DOX+Mel+USP18+3K. 1 week after tail‐vein delivery of AAV9‐USP18, mice received Mel plus compound 3K for 1 week, followed by 4 weeks of DOX before tissue collection. As before, Mel preserved cardiomyocyte size and USP18 overexpression reversed this; adding 3K restored cardiomyocyte size despite USP18 overexpression (WGA, Figure 9A). The same three‐step pattern—protection by Mel, reversal by USP18 overexpression, and rescue by 3K—was observed for fibrosis (Masson, Figure 9C), oxidative stress (DHE, Figure 9E), γH2AX (Figure 9G), Nox2 and Collagen III transcripts (Figure 9B,D,F), serum LDH (Figure 9H), and serum MDA/SOD (Figure 9I,J). Protein levels of the inflammasome marker NLRP3 and the ferroptosis markers GPX4 and FTH followed the identical trajectory: Mel lowered NLRP3 and raised GPX4/FTH, USP18 overexpression reversed these changes, and 3K restored them (Figure 9J,K). In cultured cardiomyocytes, Mel suppressed DOX‐induced DNA damage; USP18 overexpression intensified the damage, and PKM2 knockdown blunted this intensification (Figure S13A,B).

**FIGURE 9:**
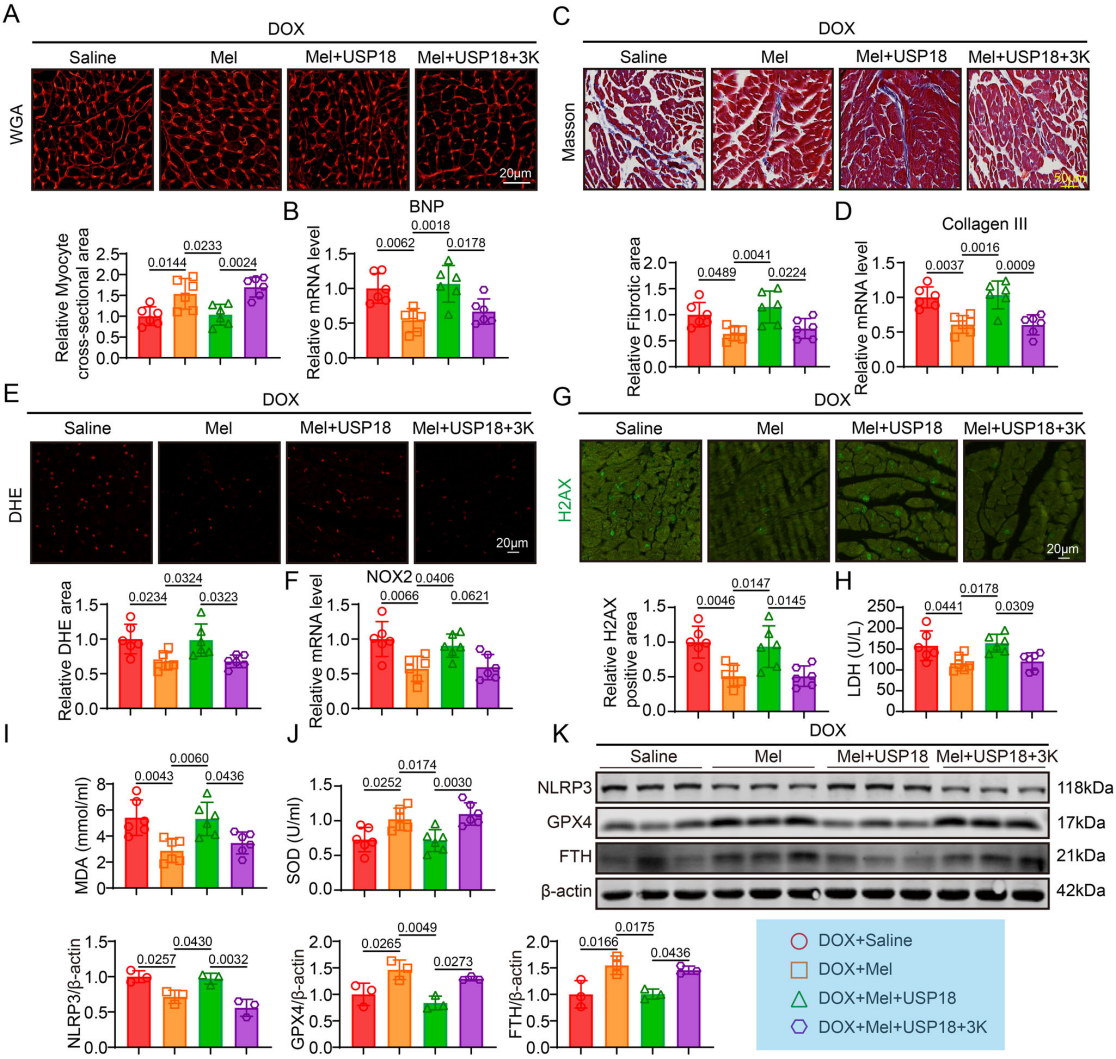
PKM2 inhibition rescues the detrimental effects of USP18 overexpression, confirming PKM2 as a downstream effector of the melatonin‐USP18 axis. (A) WGA staining and quantification of cardiomyocyte area. (B) qPCR of *BNP* mRNA. (C) Masson's trichrome staining and fibrosis quantification. (D) qPCR of *Collagen III* mRNA. (E) DHE staining and oxidative stress quantification. (F) qPCR of *Nox2*. (G) Immunofluorescence for γH2AX and quantification. (H) Serum LDH levels. (I, J) Serum SOD and MDA levels. (K) Western blot analysis of NLRP3, GPX4, and FTH.

## 4 Discussion

Combining in vivo and in vitro models with multi‐omics analyses, this work delineates an MT1/MT2‐USP18‐PKM2 signaling axis through which melatonin counteracts doxorubicin‐induced cardiotoxicity, and validates the axis in tumor‐bearing mice. Beyond clarifying DIC pathogenesis, these results inform the design of cardioprotective strategies that spare antitumor efficacy.

Melatonin can scavenge free radicals directly, but our receptor‐knockout data argue that this is not its main mode of action in DIC: losing either MT1 or MT2 markedly weakened melatonin's protection, placing receptor‐coupled cascade signaling at the center of its cardioprotective effect. Because deletion of either receptor alone only partially abolished protection, MT1 and MT2 appear to contribute to the anti‐DIC response in a non‐redundant fashion. This interpretation is consistent with the growing recognition of GPCR signaling as a determinant of cardiovascular homeostasis [21]. Ligand‐bound MT1/MT2 receptors couple to Gi protein, suppress adenylyl cyclase, and recruit β‐arrestin, thereby engaging pro‐survival routes such as PI3K/Akt and ERK1/2; the net effect is restraint of mitochondrial permeability transition pore opening and attenuation of apoptotic signaling [22]. Our data transplant this framework into the DIC setting and identify receptor signaling as the source of specificity in melatonin's cardioprotection. They also point to a practical direction for drug development: selective MT1 or MT2 agonists could retain cardioprotection while sidestepping melatonin's sedative side effect, potentially offering safer, and more druggable agents.

A key finding of this study is the identification of USP18 as a pathogenic factor in DIC and as a downstream effector of melatonin. USP18 has previously been primarily recognized for its negative feedback regulatory function in interferon signaling [23]. This study is the first to identify USP18 as a core component of the cardiomyocyte stress response to doxorubicin, where it acts as both a sensor of injury and a key effector of downstream pathological signaling. It is hypothesized that DOX‐induced DNA damage, oxidative stress, and endoplasmic reticulum stress may upregulate USP18 transcriptional expression through activation of transcription factors such as STAT1, ATF4, and others. Once upregulated, the deubiquitinating enzyme activity of USP18 may alter the homeostasis of a series of substrate proteins, thereby reshaping cell fate [24, 25, 26]. Overexpression and knockdown experiments consistently demonstrate that USP18 protein levels in cardiomyocytes are positively correlated with the severity of DIC: overexpression of USP18 is sufficient to mimic and exacerbate the cardiotoxic phenotype induced by DOX, whereas its knockdown confers significant protection. More importantly, melatonin effectively suppresses DOX‐induced USP18 upregulation through its receptor signaling, thereby blocking this pro‐injury pathway. This finding has two major implications. First, it identifies protein homeostasis regulation as a mechanism of melatonin's cardioprotection. Second, it positions USP18 as a promising target for pharmacological intervention. The development of small molecule inhibitors that specifically suppress USP18 enzymatic activity may represent an entirely new cardioprotective strategy independent of melatonin.

A second notable finding is the dual post‐translational control that USP18 exerts over PKM2. By stripping K33‐linked polyubiquitin chains, USP18 shields PKM2 from autophagic‐lysosomal degradation and sustains its abundance; by permitting K27‐linked ubiquitination, it simultaneously promotes PKM2 entry into the nucleus. The two actions cooperate to keep PKM2 both stable and nuclear, maximizing its availability as a transcriptional coactivator [27, 28]. ChIP‐qPCR data tie this mechanism to downstream pathology: in DOX‐injured cardiomyocytes, nuclear PKM2 occupies the IL1B and IL6 promoters, directly coupling USP18‐driven PKM2 nuclear import to the transcription of inflammatory and fibrotic programs. Nuclear PKM2 thus emerges as the nodal point where ubiquitin‐dependent proteostasis is converted into pathological gene expression in DIC, making the USP18‐PKM2 axis an attractive therapeutic entry point.

Pharmacological data place PKM2 downstream of the MT1/2‐USP18 axis: PKM2 inhibitors phenocopied melatonin's protection and reversed the injury caused by USP18 overexpression. Interest in PKM2 in cardiac disease is growing. Recent studies have shown, for instance, that PKM2 S‐nitrosylation promotes mitochondrial fission and drives cardiac fibrosis [20]; that PKM2 serves as a metabolic rheostat controlling pyruvate oxidation to sustain T‐cell survival [29]; and that DAPK2 phosphorylates PKM2 at Thr45 to drive its nuclear translocation and endothelial inflammation in atherosclerosis [30]. Our work extends this list by assigning PKM2 a role in chemotherapy‐induced myocardial injury and identifying USP18‐mediated deubiquitination as a new upstream regulatory mechanism. Because nuclear PKM2 dimers cooperate with transcription factors such as STAT3 to transcribe inflammatory and fibrotic genes [31], limiting PKM2 nuclear entry may be a practical way to ease DIC. Equally important for translation, experiments in the EL‐4 syngeneic lymphoma model showed that neither melatonin co‐treatment nor PKM2 inhibition blunted DOX's antitumor effect, suggesting the strategy could extend to hematological malignancies. Validation in additional anthracycline‐sensitive tumor types, including breast cancer and sarcoma, is nonetheless warranted.

Limitations requiring further investigation include: (1) while we observed elevated serum PKM2 in DOX‐treated patients, correlation analyses with cardiac function parameters and myocardial injury biomarkers were not performed due to sample size constraints, and future larger‐cohort studies are needed to validate PKM2 as a reliable clinical biomarker for DIC; (2) determination of specific USP18 deubiquitination sites on PKM2 and their effects on PKM2 structure, activity, and localization; (3) mapping nuclear PKM2 genomic binding in DIC cardiomyocytes via ChIP‐seq/CUT & Tag; (4) validation in additional tumor types (breast cancer) and with other cardiotoxic therapies; (5) optimization of clinical dosing regimens; (6) potential off‐target effects of pharmacological inhibitors; and (7) the inherent heterogeneity of human cancers not fully recapitulated in murine models; (8) several fibrosis and inflammatory readouts were derived from whole‐heart tissue homogenates, which may partially reflect contributions from non‐cardiomyocyte populations such as fibroblasts and immune cells; future studies employing cardiomyocyte‐specific transcriptomic or single‐cell approaches would further strengthen the cell‐autonomous nature of the proposed mechanism.

## 5 Conclusion

We found that melatonin antagonizes doxorubicin‐induced cardiotoxicity through the MT1/2‐USP18‐PKM2 axis. This axis links GPCR signaling, protein homeostasis, and metabolic reprogramming. These findings provide a complete evidence chain supporting melatonin's clinical investigation as a safe cardioprotective agent and establish USP18 and PKM2 as promising novel therapeutic targets for DIC, advancing the goal of maximizing anticancer efficacy while minimizing cardiotoxicity.

## Author Contributions

**Zhenyu Feng**: Data curation, Investigation, Project administration, Visualization, Writing – original draft. **Yijin Yang**: Data curation, Investigation, Project administration, Visualization, Writing – original draft. **Jie Bai**: Data curation, Investigation, Project administration, Visualization, Formal analysis, Writing – original draft. **Pengqi Sun**: Data curation, Investigation, Project administration, Visualization. **Xuyang Sun**: Data curation, Investigation, Project administration, Visualization. **Jinshu Shang**: Data curation, Investigation, Project administration, Visualization. **Yang Liu**: Data curation, Investigation, Project administration, Funding. **Zhen Ning**: Data curation, Investigation, Project administration. **Ningning Zhang**: Conception and design of research, Writing – review & editing, Supervision**. Yunlong Xia**: Conception and design of research, Writing. **Yunpeng Xie**: Conception and design of research, Writing, Formal analysis, Supervision, Funding.

## Funding

This work was supported by Natural Science Foundation of China (Grant no. 82070380); Liaoning Revitalization Talents Program (XLYC2203195); Dalian Medical University Interdisciplinary Research Cooperation Project Team Funding (JCHZ2023012); Basic Research Project for Universities of Liaoning Provincial Department of Education (LJ212610161017, LJ212510161036, LJ212510161073).

## Ethics Statement

All animal experimental procedures and protocols were approved by the Animal Care and Use Committee of Dalian Medical University (AEE21079) and complied with the NIH Guide for the Care and Use of Laboratory Animals. All efforts were made to minimize animal suffering. For experiments involving human serum samples, written informed consent was obtained from all participants, and the study protocol was reviewed and approved by the Ethics Committee of the First Affiliated Hospital of Dalian Medical University (PJ‐KS‐KY‐2025‐373). During the preparation of this manuscript, the authors used Kimi (Moonshot AI) solely for language polishing and grammar checking of the text. No AI tool was used to generate, modify, enhance, or analyze any figures, images, graphical abstracts, or other visual content. All scientific content, data, data interpretation, and conclusions are solely those of the authors, who reviewed, verified, and take full responsibility for the entire manuscript. All schematic diagrams and graphical illustrations in this manuscript were created originally by the authors using Adobe Illustrator. Data plots were generated using GraphPad Prism 10.0 and Xiantao Academic. No image generation AI tools were used. The use of all software complied with the applicable licensing terms, and no third‐party copyrighted images, logos, or trademarks were reproduced.

## Conflicts of Interest

The authors have no conflicts to interest.

## Supporting information




**Supporting File 1**: advs78431‐sup‐0001‐SuppMat.docx.

**Supporting File 2**: advs78431‐sup‐0002‐S1.docx.

**Supporting File 3**: advs78431‐sup‐0003‐S2.zip.

**Supporting File 4**: advs78431‐sup‐0004‐S3.docx.

## Data Availability

The data will be available from the corresponding author upon reasonable request.

## References

[1] F. Iacopo , C. Michela , C. Carlomaria , and C. Danielamaria , “Anthracycline‐Induced Cardiomyopathy: Risk Prediction, Prevention and Treatment,” Nature Reviews Cardiology 22 (2025): 551–563. 10.1038/s41569-025-01126-1 39875555

[2] A. Saroh , L. Christina , B. Ana , et al., “Prevention and Monitoring of Cardiac Dysfunction in Survivors of Adult Cancers,” Journal of Clinical Oncology: Official Journal of the American Society of Clinical Oncology 35, no. 8 (2017): 893–911. 10.1200/JCO.2016.70.5400 27918725

[3] L. Danl , W. Zhaov , D. Guanqiao , et al., “Doxorubicin Blocks Cardiomyocyte Autophagic Flux by Inhibiting Lysosome Acidification,” Circulation 133, no. 17 (2016): 1668–1687. 10.1161/CIRCULATIONAHA.115.017443 PMC4856587 26984939

[4] W. Kendallb , S. Vilmaa , and O. Pauloj , “Mitochondrial Determinants of Doxorubicin‐Induced Cardiomyopathy,” Circulation Research 126, no. 7 (2020): 926–941. 10.1161/CIRCRESAHA.119.314681 PMC7121924 32213135

[5] H. Joerg , L. Daniel , A. Saro , et al., “Defining Cardiovascular Toxicities of Cancer Therapies: An International Cardio‐Oncology Society (IC‐OS) Consensus Statement,” European Heart Journal 43, no. 4 (2022): 280–299. 10.1093/eurheartj/ehab674 PMC8803367 34904661

[6] A. Barbaral , D. Meenakshi , C. Lu , et al., “Cardioprotection and Safety of Dexrazoxane in Patients Treated for Newly Diagnosed T‐Cell Acute Lymphoblastic Leukemia or Advanced‐Stage Lymphoblastic Non‐Hodgkin Lymphoma: A Report of the Children's Oncology Group Randomized Trial Pediatric Oncology Group 9404,” Journal of Clinical Oncology: Official Journal of the American Society of Clinical Oncology 34, no. 8 (2016): 854–862. 10.1200/JCO.2015.60.8851 PMC4872007 26700126

[7] E. C. D. Baat , M. Renéel , H. Melissam , et al., “Mavinkurve‐Groothuis Anneliesmc, Armenian Saro. Primary Cardioprotection With Dexrazoxane in Patients with Childhood Cancer who are Expected to Receive Anthracyclines: Recommendations From the International Late Effects of Childhood Cancer Guideline Harmonization Group,” The Lancet Child & Adolescent Health 6, no. 12 (2022): 885–894. 10.1016/S2352-4642(22)00239-5 36174614

[8] G. Shom , L. Jia , G. Hao , et al., “Decline in Left Ventricular Ejection Fraction Following Anthracyclines Predicts Trastuzumab Cardiotoxicity,” JACC: Heart Failure 7, no. 9 (2019): 795–804. 10.1016/j.jchf.2019.04.014 31401102

[9] L. Yanping , H. Tianyi , R. Susan , et al., “Use of Melatonin Supplements and Risk of Type 2 Diabetes and Cardiovascular Diseases in the USA: Insights From Three Prospective Cohort Studies. The Lancet,” Diabetes & Endocrinology 12, no. 6 (2024): 404–413. 10.1016/S2213-8587(24)00096-2 PMC11500835 38710189

[10] W. Yue , Z. Wenxin , Z. Leli , et al., “Melatonin Attenuates Atherosclerotic Plaque Vulnerability Through SIRT6‐Dependent Regulation of Vascular Smooth Muscle Cells Senescence,” Redox Biology 88 (2025): 103939. 10.1016/j.redox.2025.103939 PMC12701984 41308252

[11] Z. Shengzheng , T. Wencong , D. Xianxian , et al., “Melatonin Attenuates Diabetic Cardiomyopathy by Increasing Autophagy of Cardiomyocytes via Regulation of VEGF‐B/GRP78/PERK Signaling Pathway,” Cardiovascular Diabetology 23, no. 1 (2024): 19. 10.1186/s12933-023-02078-x PMC10777497 38195474

[12] D. Zhiya , H. Man , H. Hongbin , et al., “Melatonin Attenuates Sepsis‐Induced Acute Kidney Injury by Promoting Mitophagy Through SIRT3‐Mediated TFAM Deacetylation,” Autophagy 20, no. 1 (2024): 151–165. 10.1080/15548627.2023.2252265 PMC10761103 37651673

[13] L. Ronghao , Y. Zebin , L. Wanshi , et al., “Diurnal Variation in Melatonin‐Mediated Cardiac Protection via Per2 Expression in Heart,” Journal of Pineal Research 77, no. 2 (2025): 70036. 10.1111/jpi.70036 PMC11822080 39940062

[14] G. Qingyun , W. Zixu , H. Keyu , C. Jing , D. Yulan , and C. Yaoxing , “Melatonin Ameliorates Hepatic Ferroptosis in NAFLD by Inhibiting ER Stress via the MT2/cAMP/PKA/IRE1 Signaling Pathway,” International Journal of Biological Sciences 19, no. 12 (2023): 3937–3950. 10.7150/ijbs.85883 PMC10411470 37564204

[15] T. M. Margherita , P. Claudia , R. Luigi , et al., “Melatonin MT(1) Receptors as a Target for the Psychopharmacology of Bipolar Disorder: A Translational Study,” Pharmacological Research 198 (2023): 106993. 10.1016/j.phrs.2023.106993 37972722

[16] K. Raymond , P. Vito , G. Jin , et al., “High‐Throughput Synthesis and Screening of a Cyanimide Library Identifies Selective Inhibitors of ISG15‐Specific Protease mUSP18,” Angewandte Chemie International Edition 64 (2025): 202510941. 10.1002/anie.202510941 PMC12668310 41058185

[17] J.‐Y. Lin , J.‐X. Chen , Z.‐C. Qiu , et al., “USP18 Promotes Nasopharyngeal Carcinoma Radioresistance via TRIM29 Oligomerization and Ubiquitination,” Cell Death & Differentiation 33 (2025): 10–1038. 10.1038/s41418-025-01615-3 PMC13156314 41219493

[18] W. Yang , S. Hengyao , Q. Yanzhao , et al., “PKM2 Functions as a Histidine Kinase to Phosphorylate PGAM1 and Increase Glycolysis Shunts in Cancer,” The EMBO Journal 43, no. 12 (2024): 2368–2396. 10.1038/s44318-024-00110-8 PMC11183095 38750259

[19] L. Weibo , H. Hongxia , C. Ryan , et al., “Pyruvate Kinase M2 is a PHD3‐Stimulated Coactivator for Hypoxia‐Inducible Factor 1,” Cell 145, no. 5 (2011): 732–744. 10.1016/j.cell.2011.03.054 PMC3130564 21620138

[20] L. Shanshan , Y. Danyu , Z. Yan , et al., “S‐Nitrosylation of Pyruvate Kinase Isoform 2 Drives Cardiac Fibrosis by Promoting Mitochondrial Fission,” Circulation 153, no. 5 (2026): 338–357. 10.1161/CIRCULATIONAHA.125.075903 41368700

[21] G. Pan , C. Mengying , W. Xiaolin , et al., “G Protein‐Coupled Receptor Kinase 3 Exacerbates Diabetic Heart Injuries Through Direct Phosphorylation of Cannabinoid Receptor 2 in Humans and Mice,” Circulation 152, no. 12 (2025): 882–898. 10.1161/CIRCULATIONAHA.125.073947 40772312

[22] T. Riko , A. Saki , M. Seiya , et al., “Stepwise Phosphorylation of BLT1 Defines Complex Assemblies With β‐Arrestin Serving Distinct Functions,” The FASEB Journal 37, no. 11 (2023): 23213. 10.1096/fj.202301440R 37795742

[23] C. Anne , R.‐J. Emmav , W. Iona , S. Kirbyn , U. Sylvie , and C. Michaelj , “ISGylation‐Independent Protection of Cell Growth by USP18 Following Interferon Stimulation,” The Biochemical Journal 480, no. 19 (2023): 1571–1581. 10.1042/BCJ20230301 PMC10586769 37756534

[24] F. Krista , B. Anneke , W. Robert , et al., “Trans‐eQTL Mapping Prioritises USP18 as a Negative Regulator of Interferon Response at a Lupus Risk Locus,” Nature Communications 16, no. 1 (2025): 8795. 10.1038/s41467-025-63856-7 PMC12491431 41038828

[25] M.‐F. Marta , B. Sofija , L. Z. Le Voyer Tom , et al., “A Partial Form of Inherited Human USP18 Deficiency Underlies Infection and Inflammation,” The Journal of Experimental Medicine 219, no. 4 (2022): 20211273. 10.1084/jem.20211273 PMC8908790 35258551

[26] N. Francesco , B. Rebecca , and D. S. Giannino , “Ubiquitin Choreography in Nasopharyngeal Carcinoma: USP18 Scaffolds Radioresistance,” Cell Death & Differentiation 33 (2025): 10–1038. 10.1038/s41418-025-01616-2 PMC13156269 41219492

[27] L. Kangli , Z. Xiangle , D. Dandan , et al., “Picornavirus VP2 Protein Suppresses Innate Immunity Through Selective Autophagic Degradation of IKBKE/IKKε,” Autophagy 22, no. 2 (2026): 330–350. 10.1080/15548627.2025.2597460 PMC12834145 41319264

[28] W. Zhixiang , N. Kaifan , L. Wei , et al., “ER‐Phagy Activation by AMFR Attenuates Cardiac Fibrosis Post‐Myocardial Infarction via mTORC1 Pathway,” Advanced science 12, no. 37 (2025): 4552. 10.1002/advs.202504552 PMC12499418 40673870

[29] S. Jeffj , P. Erikam , W. Jessicaf , et al., “Pyruvate Kinase Muscle 2 (PKM2) Promotes CD4 T Cell Survival by Regulating Pyruvate Oxidation During Homeostasis and Expansion,” Science Advances 12, no. 5 (2026): aec5092. 10.1126/sciadv.aec5092 PMC12857732 41616058

[30] G. Shuai , X. Long , C. Yixin , et al., “DAPK2 Regulates PKM2 Phosphorylation at Threonine 45 to Facilitate Disturbed Flow‐Induced Atherosclerosis,” Circulation 153 (2026): 10–1161. 10.1161/CIRCULATIONAHA.125.075951 41614276

[31] F. Aodi , H. Yuting , L. Qiuyu , et al., “Qishen Yiqi Dropping Pills Ameliorate Acute Myocardial Infarction by Modulating PKM2/STAT3 Pathway‐Mediated Metabolic Reprogramming in Macrophages,” Phytomedicine 151 (2026): 157784. 10.1016/j.phymed.2026.157784 41548375

