# Supplementary material for: Melatonin Mitigates Doxorubicin‐Induced Cardiotoxicity by Targeting the USP18/PKM2 Axis Without Compromising Antitumor Efficacy
Source: Adv Sci (Weinh). 2026 Oct 26:e78431. Online ahead of print. doi: 10.1002/advs.78431 (PMC13710716; doi:10.1002/advs.78431)
Supplement: Supplementary file 1 [file ADVS-9999-e78431-s001.docx]

Table 1 The primer sequences

| Gene | Forward primer (5’–3’) | Reverse primer (5’–3’) |
| --- | --- | --- |
| Mouse NOX2 | CCTCTACCAAAACCATTCGGAG | CTGTCCACGTACAATTCGTTCA |
| Mouse COL3 | CTGTAACATGGAAACTGGGGAAA | CCATAGCTGAACTGAAAACCACC |
| Mouse BNP | AGTCCTTCGGTCTCAAGGCA | CCGATCCGGTCTATCTTGTGC |
| Mouse α-SMA | CCCAGACATCAGGGAGTAATGG | TCTATCGGATACTTCAGCGTCA |
| Mouse USP18 | ACGCAGGAGTCCCTGATTTG | GGGCTGGACGAAACATCTCA |
| Mouse IL1B | CTCGACTCAGTGCGACTTCTC | CGAATGTTCATCCGCCAATTCAG |
| Mouse β-actin | GTGACGTTGACATCCGTAAAGA | GCCGGACTCATCGTACTCC |
| Human USP18 | CGAATGTTCATCCGCCAATTCAG | TCTTCAAGATCTGCCGGGGA |
| Human STAT1 | ATCAGGCTCAGTCGGGGAATA | TGGTCTCGTGTTCTCTGTTCT |
| Human β-actin | GAGAAAATCTGGCACCACACC | GGATAGCACAGCCTGGATAGCAA |
