# Supplementary material for: Melatonin Mitigates Doxorubicin‐Induced Cardiotoxicity by Targeting the USP18/PKM2 Axis Without Compromising Antitumor Efficacy
Source: Adv Sci (Weinh). 2026 Oct 26:e78431. Online ahead of print. doi: 10.1002/advs.78431 (PMC13710716; doi:10.1002/advs.78431)
Supplement: Supplementary file 2 [file ADVS-9999-e78431-s002.docx]

**Figure S1. Melatonin attenuates acute doxorubicin-induced cardiotoxicity. (A)** Schematic of acute DIC model. **(B)** Echocardiographic EF%. **(C)** WGA staining and quantification of cardiomyocyte area. **(D)** Serum LDH levels. **(E)** DHE staining and quantification. **(F)** Masson’s trichrome staining and fibrosis quantification. **(G)** qPCR analysis of *Bnp*, *Collagen III*, and *Nox2* mRNA. **(H)** Serum SOD and MDA levels.

**Figure S2. Therapeutic administration of melatonin alleviates established DIC. (A)** Experimental scheme of therapeutic melatonin intervention. **(B)** Survival curves. **(C)** HW/TL ratio. **(D)** WGA staining and cardiomyocyte cross‑sectional area quantification. **(E)** qPCR of *Bnp*. **(F)** Masson’s trichrome staining and fibrosis quantification. **(G)** qPCR of *Collagen III*. **(H)** DHE staining and quantification. **(I)** qPCR of *Nox2*. **(J)** Serum SOD and MDA. **(K)** Serum LDH.

**Figure S3. Mel inhibits DOX-induced DNA damage in cardiomyocytes. (A)** The expression of 8-OHdG in myocardial cells of each group was detected by immunofluorescence. **(B)** The expression of NOX2 in cardiomyocytes of each group was detected by qPCR.

**Figure S4. Cardiomyocyte‑specific overexpression of USP18 exacerbates DIC and abrogates melatonin’s cardioprotection.** **(A)** qPCR validation of USP18 mRNA in heart tissue. **(B)** WB was used to detect USP18 expression in the heart, liver, and lungs of mice in each group **(C)** IF examined the expression of USP18 in cardiac tissue across the groups. **(D)**  Survival curves between DOX+AAV9-Control and DOX+AAV9+USP18. **(E)** qPCR of *Nox2*. **(F)** Echocardiographic EF% and FS%. **(G)** H&E staining showing myocardial vacuolization. **(H)** Masson’s trichrome (upper) and Collagen III immunofluorescence (lower) staining. **(I)** WGA staining and quantification. **(J)** DHE staining and quantification.

**Figure S5 Knockdown of USP18 enhances the protective effect of melatonin against DOX‑induced DNA damage in cardiomyocytes. (A)** The expression of 8-OHdG in myocardial cells of each group was detected by immunofluorescence. **(B)** The expression of NOX2 in cardiomyocytes of each group was detected by qPCR.

**Figure S6 The expression of USP18 in heart, liver and lung tissues of mice with myocardial-specific knockout was detected**

**Figure S7. Melatonin alleviates DIC without affecting tumor growth in an independent syngeneic melanoma model. (A)** Experimental scheme. **(B)** Echocardiographic EF%. **(C)** Masson’s trichrome staining and fibrosis quantification. **(D)** WGA staining and cardiomyocyte area quantification. **(E)** Tumor volume measurements.

**Figure S8 USP18 affects the effect of DOX on the proliferative capacity of PANC1. (A)** The expression of PCNA in each group of cells was examined by immunofluorescence after treatment with DOX and USP18. **(B)** The expression of PCNA in each group of cells was examined by immunofluorescence after treatment with DOX and shUSP18.

**Figure S9 Doxorubicin Upregulates PKM2 via USP18 in Cardiomyocytes (A)** Co‑immunoprecipitation (co‑IP) coupled with mass spectrometry identified PKM2 as a USP18‑interacting protein. **(B, C)** Detect the expression of PKM1 and PKM2 by Western Blot after DOX-treated. **(D)** Immunofluorescence showing PKM1 (red) in cardiomyocytes. **(E)** Immunofluorescence showing PKM2 (green) co‑localized with cardiomyocyte marker α‑actinin (red) in DOX‑treated hearts. **(F)** Immunofluorescence of isolated adult mouse cardiomyocytes demonstrating DOX‑induced nuclear translocation of PKM2. **(G)** ELISA showing serum PKM2 levels in breast cancer patients before and after four cycles of liposomal doxorubicin chemotherapy. **(**H) ELISA showing serum PKM2 levels in mice after DOX treatment.

**Figure S10. PKM2 inhibitors 3K alleviate DOX-induced myocardial injury. (A)** WGA staining and quantification of cardiomyocyte area in mice treated with DOX ± PKM2 inhibitor (3K). **(B)** qPCR of *Bnp* mRNA. **(C)** Masson’s trichrome staining and fibrosis quantification. **(D)** qPCR of *Collagen III* mRNA. **(E)** DHE staining and oxidative stress quantification. **(F)** qPCR of *Nox2*. **(G)** Immunofluorescence for γH2AX and quantification. **(H)** Serum LDH levels. **(I, J)** Serum SOD and MDA levels. Parallel experiments with the PKM2 agonist TEPP‑46 showed opposite effects (data described in text).

**Figure S11 Mel and 3K inhibit DIC but do not interfere with the antitumor effects of DOX in lymphoma cells. (A)**  Masson’s trichrome staining and fibrosis quantification. **(B)** Collagen III staining and quantification. **(C)** WGA staining and cardiomyocyte cross‑sectional area quantification. **(D)** Tumor growth curves during the experimental period. **(E-G)** Tumor volume and weight at endpoint. **(H, I)** Immunohistochemical staining for PCNA and Bax and quantification.

**Figure S12. PKM2 agonists TEPP-46 aggravate DOX-induced myocardial injury. (A)** Echocardiographic EF%. **(B)** DHE staining and oxidative stress quantification. **(C)** WGA staining and quantification of cardiomyocyte area in mice treated with DOX or TEPP-46. **(D)** Masson’s trichrome staining and fibrosis quantification. **(E)** Immunofluorescence of Collagen III staining and quantification. **(F)** Immunofluorescence for γH2AX and quantification.

**Figure S13 Knockdown of PKM2 can interfere with DOX-induced cardiomyocyte DNA damage induced by USP18. (A)** The expression of 8-OHdG in myocardial cells of each group was detected by immunofluorescence. **(B)** The expression of NOX2 in cardiomyocytes of each group was detected by qPCR.
