# Supplementary material for: Melatonin Mitigates Doxorubicin‐Induced Cardiotoxicity by Targeting the USP18/PKM2 Axis Without Compromising Antitumor Efficacy
Source: Adv Sci (Weinh). 2026 Oct 26:e78431. Online ahead of print. doi: 10.1002/advs.78431 (PMC13710716; doi:10.1002/advs.78431)
Supplement: Supplementary file 3 [file ADVS-9999-e78431-s003.docx]

Table 3 The primer sequences of CHIP

| Name of Primer | | Sequence (5’ to 3’) | |
| --- | --- | --- | --- |
| il1b F1 | AGAGGCTCACTCCCTTGGAT | |  |
| il1b R1 | AACCTTGCTCCTCCTGGTTC | |  |
| il1b F2 | GAGCCTGAACCCTGCATACC | |  |
| il1b R2 | TGAGGGTGTGGGTCTCTACC | |  |
| il6 F1 | GAGAGCAAAGTCCTCACTGGG | |  |
| il6 R1 | GTGTGACTCCTTTTTGCCACC | |  |
| il6 F2 | TACAGGGAGAGGGAGCGATA | |  |
| il6 R2 | GAAGCCCACTTGGTTCAGGG | |  |
| il18 F1 | GAGCTTGCTTAGTGTGCTGC | |  |
| il18 R1 | AAAGGAAGTCTGTGGCAGGG | |  |
| il18 F2 | TGATGGTATCCGTGTGGCTT | |  |
| il18 R2 | ACCTCATTCAGGACTTCCCCT | |  |
